# CircIdh2 Attenuates Endothelial‐to‐Mesenchymal Transition and Myocardial Fibrosis and Exhibits Translational Potential

**DOI:** 10.1155/cdr/2005876

**Published:** 2026-10-08

**Authors:** Yanjia Chen, Xinai Meng, Tian Liu, Yuchen Zhang, Xingfu Huang

**Affiliations:** ^1^ Department of Anesthesiology Southern Medical University Nanfang Hospital Guangzhou China fimmu.com; ^2^ Department of Cardiology Southern Medical University Nanfang Hospital Guangzhou China fimmu.com

**Keywords:** circIdh2, circular RNA, endothelial-to-mesenchymal transition, migration, myocardial fibrosis, translational potential

## Abstract

**Background:**

Endothelial‐to‐mesenchymal transition (EndMT) contributes to myocardial fibrosis after cardiac injury. Circular RNAs (circRNAs) have been implicated in the regulation of EndMT, but whether circRNAs participate in this process through translational potential remains unclear. This study investigated the role of circIdh2 in EndMT and examined whether its coding capacity may contribute to its biological effects.

**Methods:**

Rat endothelial progenitor cells (rEPCs) were treated with TGF‐*β*1 (5 ng/mL) for 7 days to induce EndMT. The expression of circSorbs1, circIdh2, and circSntb2 was measured by qRT‐PCR. EndMT‐related markers, including VE‐cadherin, vimentin, and N‐cadherin, were detected by western blotting. Cell migration was assessed using Transwell and scratch wound healing assays. The translational potential of circIdh2 was examined using an Idh2‐IRES luciferase reporter assay, FLAG immunoprecipitation followed by western blotting, and mass spectrometry. A circIdh2 mutant construct and a rat model of acute myocardial infarction (AMI) were used to further evaluate the contribution of circIdh2 and its putative circIdh2‐derived product.

**Results:**

TGF‐*β*1 reduced the expression of circSorbs1, circIdh2, and circSntb2 in rEPCs. CircIdh2 showed the largest decrease in the initial qRT‐PCR screen and was selected before any circIdh2‐specific functional experiment. The Idh2 host gene supplied metabolic and redox context for the prespecified screen but was not used as evidence of circIdh2 function. CircIdh2 overexpression increased VE‐cadherin expression, decreased vimentin and N‐cadherin expression, and attenuated TGF‐*β*1–induced cell migration. In contrast, circIdh2 knockdown promoted EndMT‐associated marker changes and cell migration. The Idh2‐IRES reporter assay, FLAG immunoprecipitation, and mass spectrometry supported the translational potential of circIdh2 and detected a FLAG‐tagged product derived from the circIdh2 expression construct. The FLAG‐tagged circIdh2 expression construct modulated EndMT‐related markers and reduced cell migration, whereas the coding‐disrupted mutant construct did not reproduce the effects of wild‐type circIdh2. In vivo, AAV‐mediated delivery of circIdh2 or circIdh2‐aa attenuated myocardial fibrosis and partly reversed EndMT‐related marker changes after AMI.

**Conclusions:**

CircIdh2 attenuated EndMT‐associated changes in rEPCs and was associated with reduced myocardial fibrosis after AMI. These findings support the translational potential of circIdh2 and suggest that its putative circIdh2‐derived product may contribute to its effects.

## 1. Introduction

Myocardial fibrosis is an important pathological change in the progression of myocardial infarction and heart failure [1, 2]. Endothelial‐to‐mesenchymal transition (EndMT) is a dynamic process in which endothelial cells lose endothelial characteristics and acquire mesenchymal features, including enhanced migratory capacity and increased expression of mesenchymal markers [3, 4]. Lineage‐tracing and experimental studies have suggested that endothelial cells undergoing EndMT may contribute to the pool of activated fibroblasts and myofibroblasts during cardiac remodeling, although the extent of this contribution may vary among disease models 5,6. During cardiac fibrosis, endothelial cells can acquire fibroblast‐like properties under profibrotic stimuli, particularly TGF‐*β* signaling and hypoxia‐associated stress [5]. These findings support a role for EndMT in fibrotic remodeling after cardiac injury.

Circular RNAs (circRNAs) are covalently closed RNA molecules generated by back‐splicing and are generally more resistant to exonuclease‐mediated degradation than linear RNAs [6]. They participate in physiological and pathological processes by acting as transcriptional or splicing regulators, protein scaffolds, microRNA sponges, or templates for the translation of circRNA‐derived peptides or proteins [7, 8]. Several circRNAs have been reported to regulate EndMT or fibrosis‐related changes in endothelial and cardiac cells. For example, circRNA‐3302 binds miR‐135b‐5p and regulates KIT expression to promote EndMT in human umbilical vein endothelial cells (HUVECs), thereby participating in the progression of coronary artery aneurysms [9]. circZNF532 recruits TAF15 to promote PIK3CD expression, thereby promoting EndMT in human retinal microvascular endothelial cells (HRMECs) [10]. Hsa_circ_0081065 exacerbates intermittent hypoxia‐induced EndMT in HUVECs by regulating the miR‐665/HIF‐1*α* signaling axis and promoting HIF‐1*α* nuclear translocation [11]. In addition, circRNAs are involved in myocardial fibrosis. For example, circUbe3a in M2 macrophage‐derived extracellular vesicles promotes the proliferation, migration, and phenotypic transformation of cardiac fibroblasts by targeting the miR‐138‐5p/RhoC axis [12]. Circ_0002295 promotes myocardial fibrosis by targeting the miR‐1287/CXCR2 axis [13]. Some circRNAs also contain open reading frames (ORF) or internal ribosome entry sites (IRES) and can be translated into functional peptides or proteins [14, 15]. Circ_0036176 encodes Myo9a‐208, which inhibits the cyclin/Rb pathway and participates in the inhibition of cardiomyocyte proliferation [14]. However, whether circRNA‐derived peptides or proteins translated from circRNA templates can regulate EndMT remains insufficiently understood.

A previous study identified several circRNAs with predicted protein‐coding potential [16]. From that dataset, circSorbs1, circIdh2, and circSntb2 met the four eligibility criteria specified before the quantitative reverse transcription PCR (qRT‐PCR) screen: corresponding human and mouse circRNA records from orthologous host genes, a predicted ORF, detectable expression in rEPCs (Ct < 35), and a host gene linked to mitochondrial metabolism, redox control, or cardiac remodeling. Cross‐species correspondence was used only as a screening criterion and does not establish conservation of an identical back‐splice junction. CircIdh2 was chosen after the initial TGF‐*β*1 qRT‐PCR screen because it showed the largest decrease; this decision preceded all circIdh2‐specific functional experiments. IDH2 encodes a mitochondrial NADP+‐dependent isocitrate dehydrogenase involved in mitochondrial metabolism and redox homeostasis [17]. IDH2‐related mitochondrial redox regulation has been implicated in mitochondrial function and cardiac pathological remodeling [18, 19]. This host‐gene information supplied biological context for candidate selection, not evidence of circIdh2 function. Because rat endothelial progenitor cells retain endothelial‐lineage characteristics and have been shown to undergo TGF‐*β*1–induced EndMT‐like changes, they provide a responsive model for examining this process [20].

In this study, we investigated the role of circIdh2 in EndMT and myocardial fibrosis and further examined whether its putative circIdh2‐derived product may contribute to the effects of circIdh2 on EndMT‐related marker expression and cell migration.

## 2. Materials and Methods

### 2.1. Rat Endothelial Progenitor Cell Culture and EndMT Induction

Immortalized rat endothelial progenitor cells (rEPCs; #Delf‐11585) were purchased from Hefei Wanwu Biotechnology Co. Ltd. (Hefei, China; website: http://www.hfwanwu.com/). Cells were cultured in the supplier‐recommended complete medium for immortalized rEPCs (#Delf‐15862, Hefei Wanwu Biotechnology Co. Ltd.). According to the product information, 500 mL of complete medium contained 465 mL basal medium, 5 mL supplier‐provided 100× growth supplement, 25 mL fetal bovine serum, and 5 mL 100× penicillin/streptomycin. Cells were maintained at 37°C in a humidified incubator with 5% CO_2_. To induce EndMT, rEPCs were treated with recombinant TGF‐*β*1 (5 ng/mL, #HY‐P7117, MedChemExpress, Shanghai, China) for 7 days. Untreated cells cultured in parallel were used as controls.

### 2.2. Plasmid Construction and siRNA Design

All plasmids and siRNAs were synthesized by Genecfps (Jiangsu, China). The empty pCDH‐CMV‐MCS‐EF1‐GFP + Puro vector, without any inserted circRNA sequence, was used as the negative control and designated PCDH‐NC. PCDH‐circIdh2 was generated by cloning the rat circIdh2 sequence and its flanking reverse complementary sequences into the pCDH‐CMV‐MCS‐EF1‐GFP + Puro vector to promote back‐splicing and circIdh2 overexpression. A FLAG‐tagged wild‐type circIdh2 expression construct was generated using the same pCDH‐CMV‐MCS‐EF1‐GFP + Puro backbone and designated PCDH‐flag‐circIdh2. To interfere with the predicted coding capacity of circIdh2, the ATG start codon of the predicted ORF was mutated while the circIdh2 sequence and flanking reverse complementary sequences were retained. This construct was designated PCDH‐circIdh2‐mut and was used as a coding‐disrupted circIdh2 construct rather than an empty‐vector negative control. For the IRES reporter assay, the predicted 298‐bp Idh2‐IRES sequence and its mutant sequence were cloned into the pcDNA3.1‐RLuc‐luc reporter vector to generate Idh2‐IRES‐WT and Idh2‐IRES‐MUT, respectively. The siRNA sequences targeting circIdh2 were as follows: si‐circIdh2‐1 forward, 5 ^′^‐AGCUCAUCCUGCCUCACGUGGTT‐3 ^′^ and reverse, 5 ^′^‐CCACGUGAGGCAGGAUGAGCUTT‐3 ^′^; si‐circIdh2‐2 forward, 5 ^′^‐GCUCAAGCUCAUCCUGCCUCATT‐3 ^′^ and reverse, 5 ^′^‐UGAGGCAGGAUGAGCUUGAGCTT‐3 ^′^; si‐circIdh2‐3 forward, 5 ^′^‐GGCUCAAGCUCAUCCUGCCUCTT‐3 ^′^ and reverse, 5 ^′^‐GAGGCAGGAUGAGCUUGAGCCTT‐3 ^′^. The negative control siRNA sequences were forward, 5 ^′^‐UUCUCCGAACGUGUCACGUTT‐3 ^′^ and reverse, 5 ^′^‐ACGUGACACGUUCGGAGAATT‐3 ^′^.

### 2.3. Cell Transfection

Cell transfection was performed using Lipofectamine 3000 reagent (Thermo Fisher Scientific, Waltham, Massachusetts, United States). Plasmids or siRNAs and Lipofectamine 3000 were separately diluted in Opti‐MEM medium (Thermo Fisher Scientific), mixed at a 1:1 ratio, and incubated for 15 min at room temperature. The mixture was then added to rEPCs. For TGF‐*β*1–induced EndMT experiments, rEPCs were treated with TGF‐*β*1 for 7 days before plasmid transfection. Cells were collected at the indicated time points for qRT‐PCR, western blotting, Transwell migration assay, or scratch wound healing assay.

### 2.4. qRT‐PCR, Agarose Gel Electrophoresis, and Sanger Sequencing

Total RNA was extracted from cells using TRIzol reagent (Thermo Fisher Scientific). For each sample, 1 *μ*g of total RNA was reverse‐transcribed into cDNA using HiScript III RT SuperMix for qPCR (+gDNA wiper; Vazyme, Nanjing, China). qRT‐PCR was performed using ChamQ Universal SYBR qPCR Master Mix (Vazyme). *β*‐actin was used as the internal reference gene. Relative expression was calculated using the 2^−*ΔΔ*Ct^ method. Candidate selection was carried out in two stages. Before the qRT‐PCR screen, eligible candidates were required to (i) have corresponding human and mouse circRNA records from orthologous host genes, (ii) contain a predicted ORF, (iii) have a mean target Ct < 35 in untreated rEPCs, and (iv) arise from a host gene with a reported link to mitochondrial metabolism, redox control, or cardiac remodeling. These criteria yielded circSorbs1, circIdh2, and circSntb2. Their responses to TGF‐*β*1 were then measured in the initial screen. Under the prespecified prioritization rule, the candidate showing the largest decrease among those with an adjusted *p* < 0.05 was taken forward. CircIdh2 met this rule and was selected before any circIdh2‐specific gain‐ or loss‐of‐function experiment. The screen was exploratory and was not treated as evidence of circIdh2 function. For the initial screen, each candidate was normalized separately to its NC calibrator. Primer sequences are listed in Table 1. To verify the circular structure of circIdh2, divergent primers spanning the back‐splice junction were used to amplify circIdh2 from cDNA and genomic DNA (gDNA) templates. Linear Idh2 and GAPDH were amplified as controls. PCR products were separated by agarose gel electrophoresis and visualized under ultraviolet illumination. The circIdh2 PCR product was purified and subjected to Sanger sequencing by Sangon Biotech (Shanghai, China). The exon composition of circIdh2 was annotated according to the rat Idh2 genomic sequence.

**Table 1:** Primer information.

Primer name	Sequence (5 ^′^‐3 ^′^)	Length (bp)
R‐*β*‐actin‐F	AGATGACCCAGATCATGTTTGAGA	169
R‐*β*‐actin‐R	GCATGAGGGAGCGCGTAA	
rno‐circSntb2‐F	ACTGTATGCCGTGGACAAGA	211
rno‐circSntb2‐R	CCTTGGTGGTGTTCTGATGC	
rno‐circIdh2‐F	ACAGTCTGACATCCTGGCTC	198
rno‐circIdh2‐R	GCTTGAACTCTTCCACACGG	
rno‐circSorbs1‐F	TTCTTCTTCCCCAAGTCGCA	222
rno‐circSorbs1‐R	GTTTCTCTCTGGGCTGGACT	
rno‐Idh2‐F	CCAGGTCACCATCGACTCTG	197
rno‐Idh2‐R	GGCGGGGGATATTTTTGCAG	
R‐GAPDH‐F	GAAGGTCGGTGTGAACGGAT	192
R‐GAPDH‐R	ACCAGCTTCCCATTCTCAGC	

### 2.5. Fluorescence In Situ Hybridization (FISH)

FISH was performed to determine the subcellular localization of circIdh2. rEPCs were seeded on coverslips precoated with 0.01% poly‐L‐lysine (Solarbio, Beijing, China). After 24 h, cells were fixed with 4% paraformaldehyde (Solarbio) for 15 min and permeabilized with 0.5% Triton X‐100 (Solarbio) for 8 min. Cells were then treated with RNase R at 37°C for 15 min to reduce linear RNA background, and the reaction was stopped with 0.1 M glycine‐PBS. After washing with PBS, 50 *μ*L of 50 nM fluorescently labeled circIdh2‐specific probe spanning the back‐splice junction was added to each coverslip. The probe was custom‐synthesized by Sangon Biotech (Shanghai, China). Samples were denatured at 85°C for 3 min and hybridized overnight at 37°C in a humidified chamber protected from light. After hybridization, coverslips were washed three times with 2× SSC containing 50% formamide at 42°C for 15 min each, followed by two washes with 2× SSC at room temperature. Nuclei were counterstained with DAPI (Solarbio) for 5 min. Slides were mounted with antifade mounting medium and imaged under a fluorescence microscope.

### 2.6. Western Blotting

Cells or myocardial tissues were lysed in RIPA lysis buffer (Solarbio) supplemented with protease inhibitors. Lysates were centrifuged at 13,000 g for 10 min at 4°C, and the supernatants were collected. Protein concentrations were determined using a BCA protein assay kit (Solarbio). Equal amounts of protein were separated by SDS‐PAGE and transferred onto PVDF membranes (Millipore, Billerica, Massachusetts, United States). Membranes were blocked with 5% nonfat milk for 1 h at room temperature and incubated overnight at 4°C with the indicated primary antibodies. The primary antibodies used were as follows: VE‐cadherin (1:1000, #27956‐1‐AP, Proteintech, Wuhan, China), vimentin (1:1000, #ap2739b, Abcepta, Jiangsu, China), N‐cadherin (1:5000, #22018‐1‐AP, Proteintech), FLAG (1:2000, #20543‐1‐AP, Proteintech), and GAPDH (1:50000, #60004‐1‐Ig, Proteintech). After washing, membranes were incubated with species‐matched horseradish peroxidase‐conjugated secondary antibodies (Proteintech) for 1 h at room temperature. Protein bands were detected using ECL reagent (Solarbio). Band intensities were quantified using ImageJ software (Version 1.8; National Institutes of Health, Bethesda, Maryland, United States) and normalized to GAPDH. Full uncropped blot images are provided in Figure S1.

### 2.7. Transwell Migration Assay

Cell migration was assessed using Transwell chambers. Twenty‐four hours after transfection, cells were resuspended in serum‐free medium, and 1 × 10^5^ cells in 100 *μ*L were seeded into the upper chamber. The lower chamber was filled with 600 *μ*L culture medium containing 10% fetal bovine serum (Gibco, Thermo Fisher Scientific). After incubation at 37°C with 5% CO_2_ for 48 h, cells were fixed with 4% paraformaldehyde for 20 min and stained with 0.1% crystal violet (Solarbio) for 10 min. Cells remaining on the upper surface of the membrane were gently removed with a cotton swab. Migrated cells on the lower surface were imaged under a microscope. For each chamber, migrated cells were counted from randomly selected fields, and the mean value was used for statistical analysis.

### 2.8. Scratch Wound Healing Assay

Cells were seeded into six‐well plates and cultured until they reached a confluent monolayer. To minimize the effect of proliferation, cells were treated with mitomycin C (1 *μ*g/mL, MedChemExpress) for 1 h before scratching. A straight scratch was made using a sterile 200‐*μ*L pipette tip, and detached cells were removed by washing three times with PBS. Images were captured immediately after scratching and again at 24 and 48 h. Wound widths were measured using ImageJ software. Relative wound width was calculated as follows: relative wound width (%) = (wound width at the indicated 243 time point / mean wound width of the corresponding control group at 0 h) × 100%.

### 2.9. Dual‐Luciferase Reporter Assay for IRES Activity

IRESfinder was used to predict the IRES within circIdh2. The predicted 298‐bp Idh2‐IRES fragment and its mutant sequence were cloned into the pcDNA3.1‐RLuc‐luc reporter vector to generate Idh2‐IRES‐WT and Idh2‐IRES‐MUT. HEK293T cells were seeded in culture plates and transfected with the indicated reporter plasmids. After 48 h, firefly and Renilla luciferase activities were measured using the Luc‐Pair Duo‐Luciferase Assay Kit 2.0 (GeneCopoeia, Guangzhou, China) according to the manufacturer′s instructions. IRES activity was calculated as the ratio of firefly luciferase activity to Renilla luciferase activity.

### 2.10. FLAG Immunoprecipitation Followed by Western Blotting

HEK293T cells were transfected with the PCDH‐circIdh2‐FLAG‐WT plasmid. After 48 h, cells were collected and lysed on ice. Cell lysates were incubated with anti‐FLAG antibody to enrich the FLAG‐tagged product. Input lysates and immunoprecipitated samples were analyzed by western blotting using the FLAG antibody. IgG was used as a negative control for immunoprecipitation. In parallel, immunoprecipitated proteins were separated by SDS‐PAGE and stained with Coomassie Brilliant Blue. The gel region near the predicted molecular weight of the FLAG‐tagged putative circIdh2‐derived product, approximately 33 kDa, was excised for mass spectrometry analysis.

### 2.11. Mass Spectrometry Analysis

Excised gel bands were subjected to in‐gel digestion. Peptides were redissolved in nano‐LC mobile phase A containing 0.1% formic acid in water and loaded onto a nanoViper C18 precolumn (3 *μ*m, 100 Å). LC‐MS/MS analysis was performed using an EASY‐nLC 1000 nanoliter liquid chromatography system (Thermo Fisher Scientific) coupled to a Thermo Fusion mass spectrometer (Thermo Fisher Scientific) equipped with a nano Easy‐Spray ion source. Peptides were separated on a C18 reverse‐phase analytical column (PepMap RSLC, 75 *μ*m × 25 cm, C18, 2 *μ*m, 100 Å). Mobile phase B contained 80% acetonitrile and 0.1% formic acid, and the gradient increased from 5% to 38% over 60 min. The spray voltage was set to 2.0 kV, and the ion transfer tube temperature was 320°C. Data were acquired in data‐dependent acquisition mode. The full MS scan resolution was 60,000, the scan range was 300–1200 m/z, and the maximum injection time was 100 ms. Each acquisition cycle lasted 3 s. MS/MS spectra were collected for precursor ions with charge states of 2+ to 5+, with a maximum injection time of 50 ms. High‐energy collision‐induced dissociation was performed at a normalized collision energy of 28 eV, and dynamic exclusion was set to 15 s. Raw data were processed using PEAKS Studio 8.5 (Bioinformatics Solutions Inc., Waterloo, Canada). Database searching was performed against the UniProt Rattus norvegicus protein database together with a customized database containing the predicted circIdh2‐derived sequence. The search parameters were as follows: trypsin digestion, precursor mass tolerance of 10 ppm, fragment mass tolerance of 0.05 Da, and a maximum of two missed cleavages. Carbamidomethylation of cysteine was set as a fixed modification. Variable modifications included protein N‐terminal acetylation, deamidation of asparagine and glutamine, methionine oxidation, pyro‐glutamate, and pyro‐glutamine. The false discovery rate was set to 1%.

### 2.12. Animal Experiments

All animal procedures were approved by the Ethical Committee of Guangzhou Forevergen Medical Laboratory Animal Center (Approval Number IACUC‐AEWC‐F250110002, approval date: July 10, 2025) and were performed in accordance with the ARRIVE guidelines. The procedures also complied with the U.K. Animals (Scientific Procedures) Act 1986 and Directive 2010/63/EU for animal experiments. Adult male Sprague–Dawley rats weighing 200–250 g were housed under standard laboratory conditions with free access to food and water. After a 7‐day acclimation period, rats were randomly assigned to four groups: sham‐operated group, AMI model group, AMI + circIdh2‐AAV group, and AMI + circIdh2‐aa‐AAV group. The circIdh2‐AAV vector was designed to express circIdh2, whereas the circIdh2‐aa‐AAV vector was designed to express the predicted 331‐amino‐acid putative circIdh2‐derived product. Rats in the AAV‐treated groups received the corresponding AAV vector at a dose of 1 × 10^9^ PFU per rat by intramyocardial injection into the peri‐infarct region immediately after LAD ligation. Each group included three rats. Acute myocardial infarction was induced by permanent ligation of the left anterior descending coronary artery. Briefly, rats were fasted for 12 h before surgery but allowed free access to water. General anesthesia was induced with 5% isoflurane and maintained with 3% isoflurane. Adequate anesthesia was confirmed by the absence of resistance and stable respiration. Rats were placed in the supine position and endotracheally intubated. Mechanical ventilation was performed with a tidal volume of 3 mL/100 g, a respiratory rate of 80 breaths/min, and an inspiratory‐to‐expiratory ratio of 1:2. After aseptic preparation, a left thoracotomy was performed between the third and fourth intercostal spaces to expose the heart. A 4‐0 silk suture was passed around the left anterior descending coronary artery approximately 2 mm below the border between the left atrial appendage and the pulmonary conus. The ligature was tightly tied in all groups except the sham‐operated group, in which the suture was passed but not tied. Successful coronary occlusion was verified by immediate blanching of the myocardium distal to the ligature. The chest was closed in layers, and rats were kept on a warming blanket until stable respiration and heart rhythm were restored. At the endpoint, rats were deeply anesthetized with isoflurane and euthanized by exsanguination via cardiac puncture under anesthesia. Hearts were rapidly excised, rinsed in ice‐cold PBS, and processed for histological, immunofluorescence (IF), and molecular analyses.

### 2.13. HE and Masson Staining

Heart tissues were fixed in 4% paraformaldehyde, dehydrated, embedded in paraffin, and cut into 4‐*μ*m sections. Hematoxylin and eosin staining was performed using an HE staining kit (#G1120, Solarbio). Masson staining was performed using a Masson staining kit (#G1346, Solarbio). All staining procedures were carried out according to the manufacturer′s instructions. Stained sections were imaged under a light microscope.

### 2.14. IF Staining

Paraffin‐embedded myocardial sections were deparaffinized and subjected to high‐pressure antigen retrieval in EDTA buffer (pH 8.0) for 2 min. After cooling to room temperature, sections were incubated with 3% H_2_O_2_ for 10 min and blocked with 5% normal goat serum for 30 min. The sections were then incubated overnight at 4°C with primary antibodies against Collagen I (1:200, #66761‐1‐Ig, Proteintech) and TGF‐*β*1 (1:200, #81746‐2‐RR, Proteintech). After three washes with PBS, the sections were incubated with species‐matched fluorescent secondary antibodies (1:500, Proteintech) for 1 h at room temperature in the dark. Nuclei were counterstained with DAPI for 5 min. Slides were mounted with antifade mounting medium and imaged under a fluorescence microscope. Fluorescence intensity was quantified using ImageJ software from randomly selected fields.

### 2.15. Statistical Analysis

Statistical analysis was performed using GraphPad Prism 7.0 (GraphPad Software, San Diego, California, United States). Data are presented as mean ± SD. Cell experiments were repeated in three independent biological experiments, and animal experiments included three rats per group. The *n* value and statistical test used for each experiment are stated in the corresponding figure legends. Data normality was assessed using the Shapiro–Wilk test before statistical comparison. For the initial candidate screen in Figure 1A, two‐way analysis of variance followed by Šídák′s multiple‐comparisons test was used to compare NC and TGF‐*β*1 within each circRNA. For other comparisons between two independent groups, an unpaired Student′s *t*‐test was used. For comparisons among three or more independent groups, one‐way analysis of variance followed by Tukey′s post hoc test was used. A value of *p* < 0.05 was considered statistically significant.

**Figure 1:**
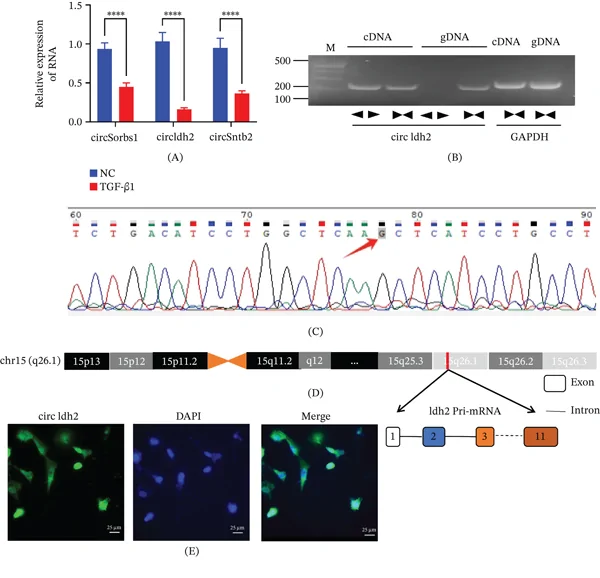
CircIdh2 expression was downregulated in TGF‐*β*1–induced rEPCs. (A) rEPCs were treated with TGF‐*β*1 (5 ng/mL) for 7 days. qRT‐PCR analysis of circSorbs1, circIdh2, and circSntb2. (B) Agarose gel electrophoresis was used to detect the PCR products of circIdh2, Idh2, and GAPDH using cDNA and gDNA templates. (C) Sanger sequencing was used to detect the back‐splice junction of circIdh2. (D) Rat circIdh2 was formed by back‐splicing of exons 1, 2, 3, and 11 from Idh2 pre‐mRNA; the human IDH2 locus is shown at chr15(q26.1). (E) FISH showed that circIdh2 was mainly localized in the cytoplasm. Scale bars, 25 *μ*m. Data are mean ± SD; *n* = 3 independent biological experiments. Two‐way ANOVA followed by Šídák′s multiple‐comparisons test was used to compare NC and TGF‐*β*1 within each circRNA; ∗∗∗∗*p* < 0.0001.

## 3. Results

### 3.1. CircIdh2 Expression Was Downregulated in TGF‐*β*1–Induced rEPCs

Table 2 lists the rat circRNAs considered and the corresponding human and mouse circRNA records from orthologous host genes. The four eligibility criteria yielded circSorbs1, circIdh2, and circSntb2; the complete initial screening data are provided in Table S1. TGF‐*β*1 reduced all three candidates (adjusted *p* < 0.0001 for each comparison). The mean TGF‐*β*1/NC ratios were 0.48 for circSorbs1, 0.16 for circIdh2, and 0.39 for circSntb2 (Figure 1A). CircIdh2 therefore showed the largest decrease and was selected under the prespecified prioritization rule before any circIdh2‐specific functional experiment. This screen was used to prioritize a candidate, not as evidence of biological importance. Agarose gel electrophoresis indicated that circIdh2 was amplified from cDNA but not from gDNA (Figure 1B), and Sanger sequencing confirmed its back‐splice junction (Figure 1C). Rat circIdh2 was generated from exons 1, 2, 3, and 11 of Idh2 pre‐mRNA; the corresponding human IDH2 locus is at chr15(q26.1) (Figure 1D). FISH, performed after candidate prioritization, showed that circIdh2 was predominantly cytoplasmic (Figure 1E). These experiments verified the circular structure and subcellular localization of circIdh2 and confirmed its downregulation in TGF‐*β*1–treated rEPCs.

**Table 2:** CircRNA information.

Rat circRNA locus (source dataset)	Host gene	Corresponding human circRNA record(s)	Corresponding mouse circRNA record(s)
chr14:44496991|44498906	Ugdh	hsa_circ_0126264	None
chr1:259396273|259437470	Sorbs1	hsa_circ_0094610; hsa_circ_0094609	mmu_circ_0007771; mmu_circ_0007772
chr1:141877382|141880461	Idh2	hsa_circ_0057970	mmu_circ_0001587
chr9:81258380|81275269	Tns1	hsa_circ_0004690	None
chr19:39176540|39195019	Sntb2	hsa_circ_0005733; hsa_circ_0105819; hsa_circ_0040039	mmu_circ_0014531

### 3.2. CircIdh2 Overexpression Attenuated TGF‐*β*1–Induced EndMT Changes in rEPCs

To investigate the function of circIdh2, rEPCs were treated with or without TGF‐*β*1 for 7 days and then transfected with PCDH‐NC or PCDH‐circIdh2. Compared with the PCDH‐NC group, TGF‐*β*1 treatment reduced circIdh2 expression, whereas PCDH‐circIdh2 restored circIdh2 expression in TGF‐*β*1–treated cells (Figure 2A). Western blotting indicated that TGF‐*β*1 decreased the endothelial marker VE‐cadherin and increased the mesenchymal markers vimentin and N‐cadherin. Compared with the TGF‐*β*1 + PCDH‐NC group, PCDH‐circIdh2 partly reversed these changes, with increased VE‐cadherin and decreased vimentin and N‐cadherin expression (Figure 2B). Transwell assays and scratch wound analysis indicated that TGF‐*β*1 increased the number of migrated cells and reduced relative wound width, reflecting enhanced migratory activity. In contrast, PCDH‐circIdh2 reduced the number of migrated cells and preserved a larger relative wound width, indicating attenuation of the TGF‐*β*1–induced migratory response (Figure 2C–E).

**Figure 2:**
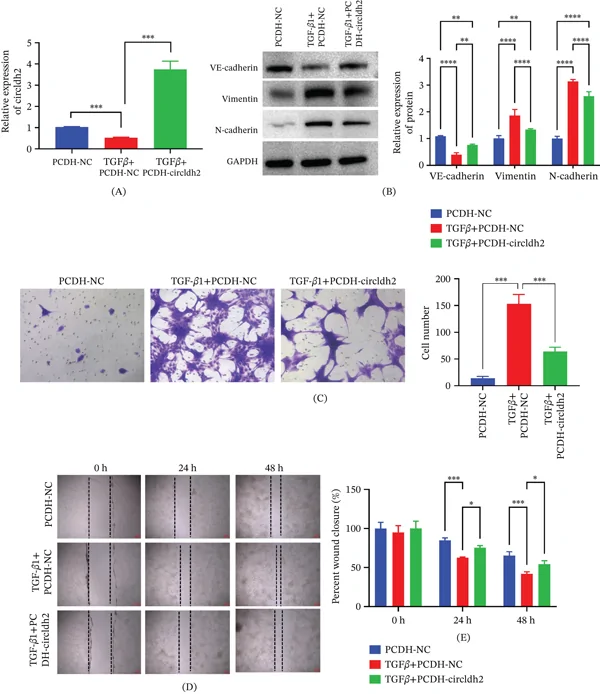
circIdh2 attenuated TGF‐*β*1–induced EndMT changes in rEPCs. rEPCs were treated with or without TGF‐*β*1 (5 ng/mL) for 7 days, and then transfected with PCDH‐NC or PCDH‐circIdh2. (A) qRT‐PCR analysis of circIdh2 expression after transfection for 24 h. (B) Western blot analysis of EndMT‐related proteins (VE‐cadherin, vimentin, and N‐cadherin) after transfection for 48 h. (C) Transwell assay was performed to assess cell migration after transfection for 24 h. (D) Representative scratch wound healing images at 0, 24, and 48 h. Scale bars, 200 *μ*m. (E) Quantification of relative wound width (% of PCDH‐NC at 0 h). Data are mean ± SD; *n* = 3 independent biological experiments; one‐way ANOVA; ∗*p* < 0.05, ∗∗*p* < 0.01, ∗∗∗*p* < 0.001, and ∗∗∗∗*p* < 0.0001.

### 3.3. Knockdown of CircIdh2 Promoted EndMT Changes in rEPCs

In addition, circIdh2 was knocked down to further evaluate its role in rEPCs. Compared with the si‐NC group, siRNAs targeting circIdh2 significantly inhibited the expression of circIdh2, among which si‐circIdh2‐1 showed the strongest knockdown efficiency and was selected for subsequent experiments (Figure 3A). Compared with the si‐NC group, circIdh2 knockdown decreased VE‐cadherin expression and increased vimentin and N‐cadherin expression (Figure 3B). Transwell assays showed that circIdh2 knockdown increased the number of migrated cells, and scratch wound analysis showed a lower relative wound width at 48 h, together indicating enhanced cell migration after circIdh2 knockdown (Figure 3C–E). These findings indicated that loss of circIdh2 promoted EndMT‐associated marker changes and migratory behavior in rEPCs.

**Figure 3:**
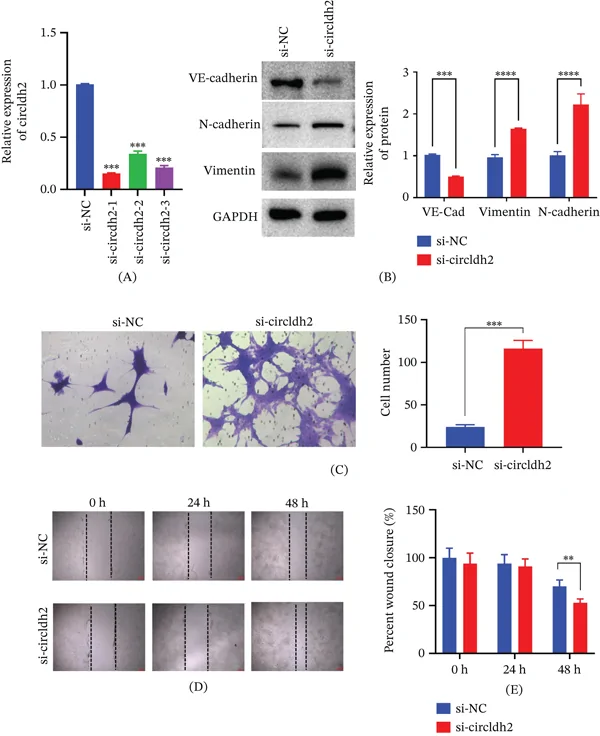
Knockdown of circIdh2 promoted EndMT changes in rEPCs. rEPCs were transfected with si‐NC or siRNAs targeting circIdh2. (A) qRT‐PCR analysis of circIdh2 expression after transfection for 24 h. (B) Western blot analysis of EndMT‐related proteins (VE‐cadherin, vimentin, and N‐cadherin) after transfection for 48 h. (C) Cell migration was assessed using a Transwell assay after transfection for 24 h. (D) Representative scratch wound healing images were obtained at 0, 24, and 48 h. Scale bars, 200 *μ*m. (E) Quantification of relative wound width (% of si‐NC at 0 h). Data are mean ± SD; *n* = 3 independent biological experiments; Student′s *t*‐test was used for two‐group comparisons and one‐way ANOVA for multiple‐group comparisons; ∗∗*p* < 0.01, ∗∗∗*p* < 0.001, and ∗∗∗∗*p* < 0.0001.

### 3.4. CircIdh2 Showed Translational Potential and Generated a Detectable FLAG‐Tagged Product in an Overexpression System

IRESfinder was used to analyze the IRES information of circIdh2. A luciferase reporter assay was then performed to examine IRES activity. The wild‐type Idh2‐IRES increased luciferase activity, whereas mutation of the IRES reduced this activity, supporting the translational potential of circIdh2 (Figure 4A). Subsequently, the PCDH‐circIdh2‐FLAG‐WT plasmid was constructed, and the expression of FLAG was detected by co‐immunoprecipitation followed by western blotting. FLAG was detected in both the input and IP samples (Figure 4B). The products of coimmunoprecipitation were then subjected to SDS‐PAGE and stained with Coomassie Brilliant Blue, and the target region near the predicted molecular weight of the product was excised for mass spectrometry analysis (Figure 4C). Mass spectrometry identified the peptide sequence SGTSIGSLTTWWLR, which matched the predicted putative circIdh2‐derived product (Figure 4D). These data supported the translational potential of circIdh2 and indicated the presence of a detectable FLAG‐tagged product in the circIdh2 overexpression system.

**Figure 4:**
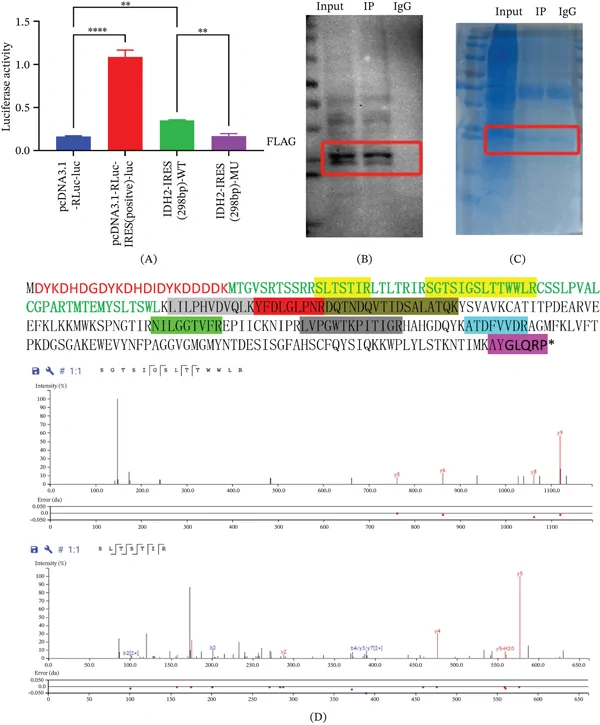
circIdh2 showed translational potential and a FLAG‐tagged product was detected in an overexpression system. (A) Luciferase activity of the Idh2‐IRES element and its mutant form. (B) Co‐IP followed by western blotting was used to detect FLAG‐tagged protein expression, and (C) Coomassie Brilliant Blue staining showed the protein region selected for mass spectrometry. (D) Mass spectrometry results showing the peptide sequence corresponding to the putative circIdh2‐derived product and the representative MS/MS spectrum. Data are mean ± SD; *n* = 3 independent experiments; one‐way ANOVA; ∗∗*p* < 0.01 and ∗∗∗∗*p* < 0.0001.

### 3.5. The FLAG‐Tagged CircIdh2 Construct Affected EndMT‐Related Markers and Cell Migration in rEPCs

To evaluate the functional effect of the FLAG‐tagged circIdh2 construct, rEPCs were transfected with PCDH‐NC or PCDH‐flag‐circIdh2. qRT‐PCR revealed a significant upregulation of circIdh2 expression in the PCDH‐flag‐circIdh2 group compared with the PCDH‐NC group (Figure 5A). Western blotting showed increased FLAG expression in the PCDH‐flag‐circIdh2 group (Figure 5B). Compared with the PCDH‐NC group, the PCDH‐flag‐circIdh2 group showed increased VE‐cadherin expression, decreased vimentin and N‐cadherin expression, fewer migrated cells in the Transwell assay, and a larger relative wound width in the scratch wound healing assay at 48 h, consistent with reduced migratory activity (Figure 5C–F). These results indicated that the FLAG‐tagged circIdh2 expression construct modulated EndMT‐related markers and reduced migratory activity in rEPCs.

**Figure 5:**
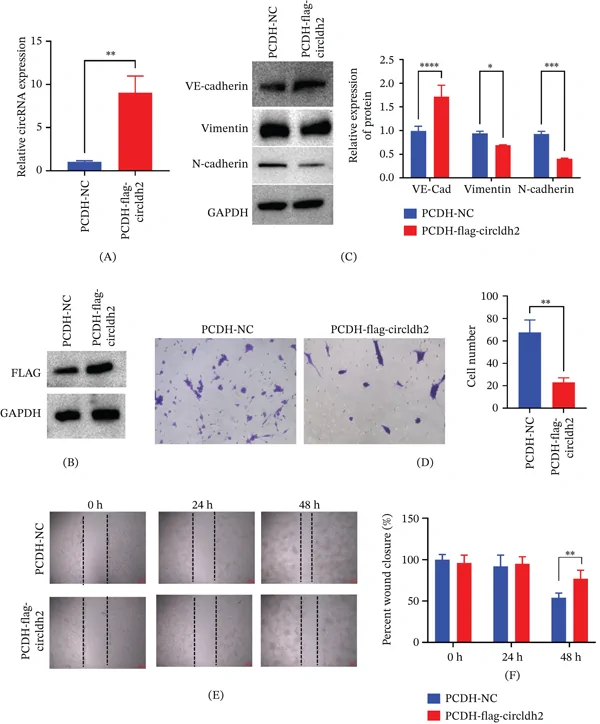
The FLAG‐tagged circIdh2 construct affected EndMT‐related markers and cell migration in rEPCs. rEPCs were transfected with PCDH‐NC or PCDH‐flag‐circIdh2. (A) qRT‐PCR analysis of circIdh2 expression after transfection for 24 h. (B) Western blot analysis of FLAG expression after transfection for 48 h. (C) Western blot analysis of EndMT‐related proteins (VE‐cadherin, vimentin, and N‐cadherin) after transfection for 48 h. (D) Cell migration was examined by Transwell assay after transfection for 24 h. (E) Representative images from the scratch wound healing assay at 0, 24, and 48 h. Scale bar, 200 *μ*m. (F) Quantification of relative wound width (% of PCDH‐NC at 0 h). Data are mean ± SD; *n* = 3 independent biological experiments; unpaired Student′s *t*‐test; ∗*p* < 0.05, ∗∗*p* < 0.01, ∗∗∗*p* < 0.001, and ∗∗∗∗*p* < 0.0001.

### 3.6. The CircIdh2 Mutant Did Not Reproduce the Effects of Wild‐Type CircIdh2 on EndMT‐Related Changes

To examine whether the predicted coding capacity contributed to the effects of circIdh2, rEPCs were transfected with PCDH‐NC, PCDH‐circIdh2, or the coding‐disrupted construct PCDH‐circIdh2‐mut. Compared with PCDH‐NC, PCDH‐circIdh2 increased VE‐cadherin expression and decreased vimentin and N‐cadherin expression (Figure 6). PCDH‐circIdh2‐mut showed weaker effects than wild‐type circIdh2 and did not fully reproduce the wild‐type expression pattern (Figure 6A). In the Transwell assay, PCDH‐circIdh2 markedly reduced the number of migrated cells, whereas PCDH‐circIdh2‐mut showed a migratory level closer to that of PCDH‐NC. Similarly, scratch wound analysis showed that wild‐type circIdh2 maintained a larger relative wound width at 48 h, whereas PCDH‐circIdh2‐mut remained comparable with PCDH‐NC and failed to reproduce the antimigratory effect of wild‐type circIdh2 (Figure 6B,C). These results suggest that disruption of the predicted start codon attenuated part of the circIdh2‐associated effects, supporting the possibility that the predicted translation‐related activity may contribute to EndMT‐related marker expression and cell migration.

**Figure 6:**
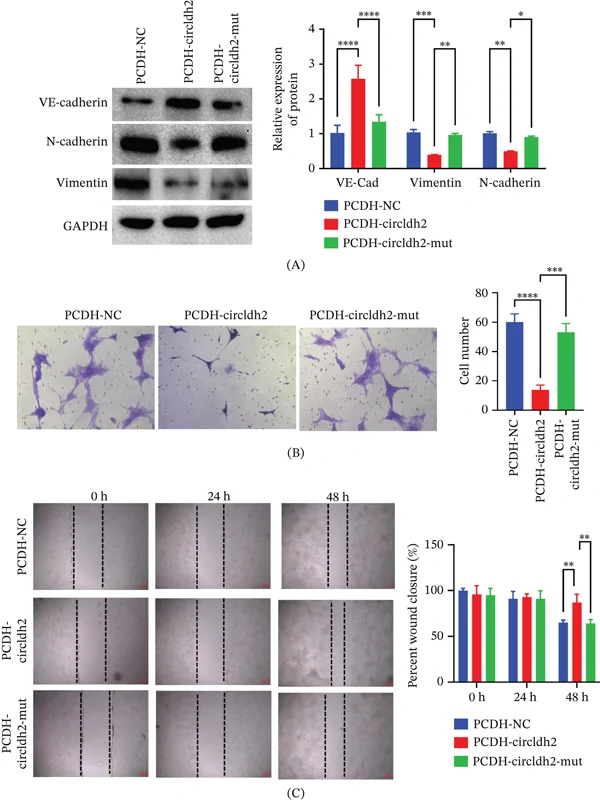
The circIdh2 mutant did not reproduce the effects of wild‐type circIdh2 on EndMT‐related markers and cell migration. rEPCs were transfected with PCDH‐NC, PCDH‐circIdh2, or PCDH‐circIdh2‐mut. (A) Western blot analysis of EndMT‐related proteins (VE‐cadherin, vimentin, and N‐cadherin) after transfection for 48 h. (B) Cell migration was assessed using a Transwell assay after transfection for 24 h. (C) Representative scratch wound healing images at 0, 24, and 48 h, with quantification of relative wound width (% of PCDH‐NC at 0 h). Scale bar, 200 *μ*m. Data are mean ± SD; *n* = 3 independent biological experiments; one‐way ANOVA; ∗*p* < 0.05, ∗∗*p* < 0.01, ∗∗∗*p* < 0.001, and ∗∗∗∗*p* < 0.0001.

### 3.7. AAV‐Mediated Delivery of CircIdh2 or CircIdh2‐aa Attenuated Myocardial Fibrosis and EndMT‐Related Marker Changes In Vivo

Furthermore, the in vivo relevance of circIdh2 and circIdh2‐aa was examined in a rat model of AMI. HE staining revealed that, in the sham group, cardiomyocytes were neatly aligned, with no obvious swelling, degeneration, or necrosis. In the model group, myocardial disorganization, inflammatory infiltration, and injury‐related histological changes were observed. After AAV‐mediated delivery of circIdh2 or circIdh2‐aa, these histological changes were partly improved (Figure 7A). Masson staining indicated that myocardium in the sham group retained an organized structure, whereas the model group exhibited increased collagen deposition and disrupted myocardial architecture. CircIdh2‐AAV and circIdh2‐aa‐AAV reduced fibrotic staining and improved tissue organization (Figure 7B). IF showed that Collagen I and TGF‐*β* signals were increased in the model group and decreased after circIdh2‐AAV or circIdh2‐aa‐AAV administration (Figure 7C). Western blot analysis further revealed that vimentin and N‐cadherin were upregulated in the model group, whereas VE‐cadherin was downregulated. These changes were partly reversed by circIdh2‐AAV and circIdh2‐aa‐AAV treatment (Figure 7D). Together, these in vivo data indicated that circIdh2 and circIdh2‐aa were associated with reduced myocardial fibrosis and attenuation of EndMT‐related marker changes after AMI.

**Figure 7:**
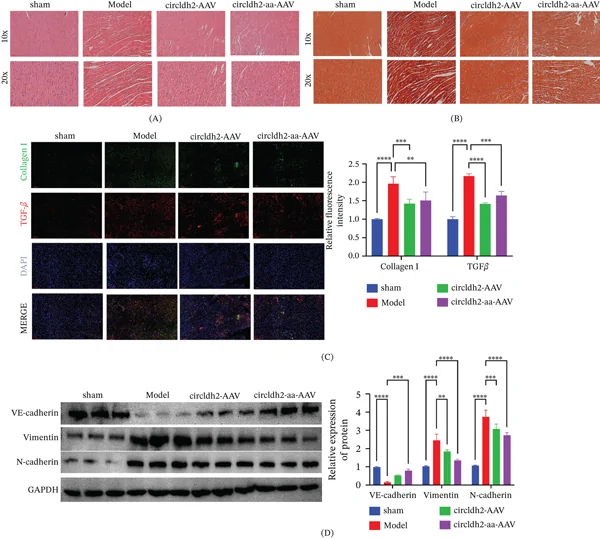
AAV‐mediated delivery of circIdh2 or circIdh2‐aa attenuated myocardial fibrosis and EndMT‐related marker changes in vivo. (A) HE staining of myocardial infarction tissues from the sham, model, circIdh2‐AAV, and circIdh2‐aa‐AAV groups. (B) Masson staining of MI tissues. (C) IF detection and quantification of fibrotic markers (Collagen I and TGF‐*β*) in myocardial tissues. Scale bars, 100 *μ*m. (D) Western blot analysis of EndMT‐related proteins (VE‐cadherin, vimentin, and N‐cadherin). Data are mean ± SD; *n* = 3 rats per group; one‐way ANOVA; ∗∗*p* < 0.01, ∗∗∗*p* < 0.001, and ∗∗∗∗*p* < 0.0001.

## 4. Discussion

This study supports a role for circIdh2 in EndMT‐associated phenotypic regulation and myocardial fibrotic remodeling. In TGF‐*β*1–treated rEPCs, reduced circIdh2 expression accompanied the acquisition of an EndMT‐like phenotype, whereas circIdh2 restoration was associated with preservation of VE‐cadherin expression, lower vimentin and N‐cadherin expression, and reduced migratory activity. Beyond this RNA‐level effect, the Idh2‐IRES reporter assay, FLAG immunoprecipitation, western blotting, and mass spectrometry supported the translational potential of circIdh2 and the presence of a detectable FLAG‐tagged putative circIdh2‐derived product in the overexpression system. The mutant construct further suggested that the predicted translation‐related activity may contribute to part of the circIdh2‐related effects. In the AMI rat model, AAV‐mediated delivery of circIdh2 or circIdh2‐aa was associated with reduced myocardial fibrosis and partial normalization of EndMT‐related markers. Together, these findings place circIdh2 at the intersection of circRNA‐mediated regulation, translational potential, EndMT‐associated phenotypic change, and myocardial fibrotic remodeling.

EndMT is characterized by loss of endothelial features and acquisition of mesenchymal traits, including increased migration and altered expression of endothelial and mesenchymal markers [21–23]. This process has been implicated in fibrotic remodeling in cardiovascular and other pathological settings [24–26]. In the present study, TGF‐*β*1 induced an EndMT‐like phenotype in rEPCs, as reflected by reduced VE‐cadherin, increased vimentin and N‐cadherin, and enhanced migration [5, 26]. The coordinated changes in marker expression and migratory behavior indicate that circIdh2 is linked to a broader phenotypic program rather than to a single EndMT‐related marker [23]. This model is supported by previous evidence showing that EPCs can undergo TGF‐*β*1–induced transition toward a mesenchymal phenotype [27]. Meanwhile, rEPCs retain greater phenotypic plasticity than mature endothelial cells [28]. Therefore, the cellular changes observed here indicate an EndMT‐like phenotype in the rEPC model, whereas further validation in mature endothelial cells is needed to define their broader relevance to vascular EndMT. The contribution of EndMT to tissue remodeling appears to be context‐dependent and may vary according to disease stage, tissue microenvironment, and the degree of endothelial plasticity [29]. In fibrotic cardiovascular remodeling, several experimental studies have shown that attenuation of excessive EndMT is accompanied by reduced fibrosis or improved cardiac function [2, 30]. Recombinant human BMP‐7 was reported to inhibit EndMT and reduce cardiac fibrosis in mouse models of pressure overload and chronic allograft rejection [5]. In myocardial infarction‐related fibrosis, miR‐146a‐5p overexpression attenuated cardiac fibrosis by suppressing EndMT‐related changes in cardiac microvascular endothelial cells [1]. Dapagliflozin also reduced EndMT in a nondiabetic heart failure model and was associated with improved myocardial perivascular fibrosis and cardiac dysfunction through SIRT1‐mediated regulation of endothelial Notch1 signaling [2]. These findings suggest that aberrant EndMT can contribute to fibrotic cardiac remodeling under defined pathological conditions. In this context, the inhibitory effects of circIdh2 on TGF‐*β*1–induced EndMT‐like changes in rEPCs, together with the reduction of myocardial fibrosis after AMI, support a possible role for circIdh2 in modulating pathological endothelial phenotypic transition during cardiac remodeling. Consistent with the involvement of circRNAs in endothelial phenotypic regulation, circZNF532 was reported to facilitate EndMT in diabetic retinopathy by recruiting TAF15 to stabilize PIK3CD, and hsa_circ_0081065 was shown to aggravate intermittent hypoxia‐induced EndMT through the miR‐665/HIF‐1*α* axis [10, 11]. These examples support the view that circRNAs can regulate EndMT through disease‐ and context‐specific mechanisms, providing a relevant framework for the circIdh2‐related phenotypic changes observed in this study.

Selection of circIdh2 was an exploratory prioritization step rather than evidence of biological importance. circSorbs1, circIdh2, and circSntb2 first met the four eligibility criteria, and circIdh2 was then chosen because it showed the largest TGF‐*β*1–associated decrease in the initial screen. This decision was made before the circIdh2‐specific functional experiments. The established role of Idh2 in mitochondrial metabolism and redox regulation supplied context for the initial screen, but host‐gene function cannot be assigned to a circRNA. We therefore evaluated circIdh2 through independent gain‐ and loss‐of‐function experiments. Idh2 encodes mitochondrial NADP+‐dependent isocitrate dehydrogenase, an enzyme involved in mitochondrial metabolism and redox homeostasis [19]. IDH2‐related redox regulation has been linked to mitochondrial function, oxidative stress control, and cardiac pathological remodeling [31]. Recent evidence further supports IDH2 as an important component of mitochondrial antioxidative defense in cardiomyocytes, whereas TGF‐*β*‐related signaling has been closely associated with repair, remodeling, and fibrosis after cardiac injury [32]. Recent work also reported that circIDH2 regulates adipogenic differentiation in porcine preadipocytes through the miR‐193a‐5p/RASGRP4 axis [33]. Although adipogenesis and EndMT are distinct biological processes, both involve changes in cell‐state regulation. Together with the current functional data, these findings support a possible role for circIdh2 in endothelial phenotypic transition and myocardial fibrotic remodeling.

The downstream mechanisms by which circIdh2 and its putative circIdh2‐derived product affect EndMT‐associated changes remain to be defined. TGF‐*β*/Smad signaling is a central pathway in EndMT and can cooperate with transcriptional regulators such as Snail, Slug, and Twist to promote mesenchymal gene expression [34, 35]. Notch and Wnt/*β*‐catenin signaling also participate in endothelial phenotypic transition and can interact with TGF‐*β*‐related pathways in a context‐dependent manner [36]. Because the observed changes involved preservation of an endothelial marker, suppression of mesenchymal markers, and reduced migration, circIdh2 and/or its putative circIdh2‐derived product may act upstream of, or in parallel with, transcriptional programs that govern endothelial identity and mesenchymal acquisition. Mitochondrial redox status can intersect with EndMT‐associated signaling, but the present study did not test whether circIdh2 acts through that route [19]. Direct measurements of mitochondrial and redox signaling will be needed to determine whether either circIdh2 or its putative product affects this pathway.

The present findings also extend previous observations that several circRNA‐derived peptides or proteins participate in disease‐related biological processes [37–39]. Recent immunopeptidomics work has identified naturally presented back‐splice junction‐spanning circRNA‐derived peptides, supporting the broader concept that circRNA‐derived products can be detected at the peptide level [37, 40]. In this study, the Idh2‐IRES reporter assay supported the activity of the predicted IRES sequence, and FLAG immunoprecipitation combined with western blotting and mass spectrometry supported the presence of a FLAG‐tagged putative circIdh2‐derived product in the overexpression system. The mutant construct suggested that the predicted translation‐related activity may contribute to the effects observed with wild‐type circIdh2. However, these experiments do not fully separate the RNA‐dependent effects of circIdh2 from those of its putative circIdh2‐derived product. The PCDH‐flag‐circIdh2 construct increases circIdh2 expression while allowing detection of the FLAG‐tagged product, and disruption of the predicted coding sequence does not exclude all RNA‐level effects. The data therefore support the translational potential of circIdh2 and suggest that the putative product may contribute to the observed effects, but they do not establish endogenous production. Junction‐specific antibodies or targeted mass spectrometry will be required to verify whether this product is generated endogenously.

The in vivo findings are consistent with the proposed role of circIdh2 in fibrotic remodeling. In the AMI model, circIdh2‐AAV and circIdh2‐aa‐AAV were associated with improved myocardial tissue organization, reduced fibrotic staining, lower Collagen I and TGF‐*β* signals, and partial normalization of VE‐cadherin, vimentin, and N‐cadherin expression. These findings connect the in vitro EndMT‐associated observations with myocardial fibrosis after cardiac injury [26, 32]. Because cardiac functional outcomes were not assessed, these findings support reduced fibrotic remodeling and provide a basis for further functional validation.

The study also has limitations. The in vitro experiments were performed in rEPCs, which provide a useful model for examining endothelial‐lineage plasticity and TGF‐*β*1–induced EndMT‐like changes. However, because EPCs have greater intrinsic plasticity than mature endothelial cells, these findings should be interpreted as EndMT‐like phenotypic changes in an rEPC model. Future validation in mature human endothelial cells, such as HUVECs, HMECs, or HAECs, would help define the broader relevance of circIdh2 to vascular EndMT. The in vivo experiments focused on histological, IF, and protein marker changes, whereas cardiac functional measurements, such as echocardiographic parameters, were not included. Future studies incorporating these assessments would further clarify the functional significance of the observed reduction in fibrotic remodeling. The present study also did not directly examine downstream signaling events, including TGF‐*β*/Smad activation, Snail/Slug/Twist expression, Notch signaling, Wnt/*β*‐catenin signaling, or mitochondrial redox status. In addition, although the data support the translational potential of circIdh2 and suggest that its putative circIdh2‐derived product may contribute to its effects, the relative contributions of RNA‐level regulation and the putative circIdh2‐derived product remain to be defined in future studies.

## 5. Conclusions

CircIdh2 attenuated TGF‐*β*1–induced EndMT‐associated marker changes and cell migration in rEPCs. The Idh2‐IRES reporter assay, FLAG immunoprecipitation, mass spectrometry, and mutant construct analyses supported the translational potential of circIdh2 and suggested that its putative circIdh2‐derived product may contribute to these effects. In AMI rats, AAV‐mediated delivery of circIdh2 or circIdh2‐aa was associated with reduced myocardial fibrosis and partial normalization of EndMT‐related markers. These findings support a regulatory role for circIdh2 in EndMT‐associated remodeling and provide a basis for further studies on circRNA translational potential in myocardial fibrosis.

## Author Contributions

Yanjia Chen, Xinai Meng, and Tian Liu performed experiments. Yanjia Chen and Xingfu Huang analyzed the results. Xingfu Huang revised the manuscript. Yanjia Chen wrote the paper. Xingfu Huang revised the manuscript.

## Funding

This work was supported by the Guangdong Basic and Applied Basic Research Foundation (2022A1515110976) and the President Foundation of Nanfang Hospital, Southern Medical University (2023A012).

## Disclosure

All authors approved the final manuscript.

## Ethics Statement

All animal procedures were approved by the Ethical Committee of Guangzhou Forevergen Medical Laboratory Animal Center (Approval Number IACUC‐AEWC‐F250110002, date 2025‐7‐10) and were conducted in strict accordance with the ARRIVE guidelines. All animal experiments comply with the U.K. Animals (Scientific Procedures) Act, 1986 and associated guidelines, EU Directive 2010/63/EU for animal experiments.

## Consent

The authors have nothing to report.

## Conflicts of Interest

The authors declare no conflicts of interest.

## Supporting Information

Additional supporting information can be found online in the Supporting Information section.

## Supporting information


**Supporting Information 1** Table S1: Prespecified eligibility information and initial qRT‐PCR screening results for candidate circRNAs.

**Supporting Information 2** Figure S1: Full uncropped blot images for Figures 1, 2, 3, 4, 5, 6, and 7.

## Data Availability

The data that support the findings of this study are available from the corresponding author upon reasonable request.
