# Supplementary material for: CircIdh2 Attenuates Endothelial‐to‐Mesenchymal Transition and Myocardial Fibrosis and Exhibits Translational Potential
Source: Cardiovasc Ther. 2026 Oct 8;2026:2005876. doi: 10.1155/cdr/2005876 (PMC13647212; doi:10.1155/cdr/2005876)

Fig 1B

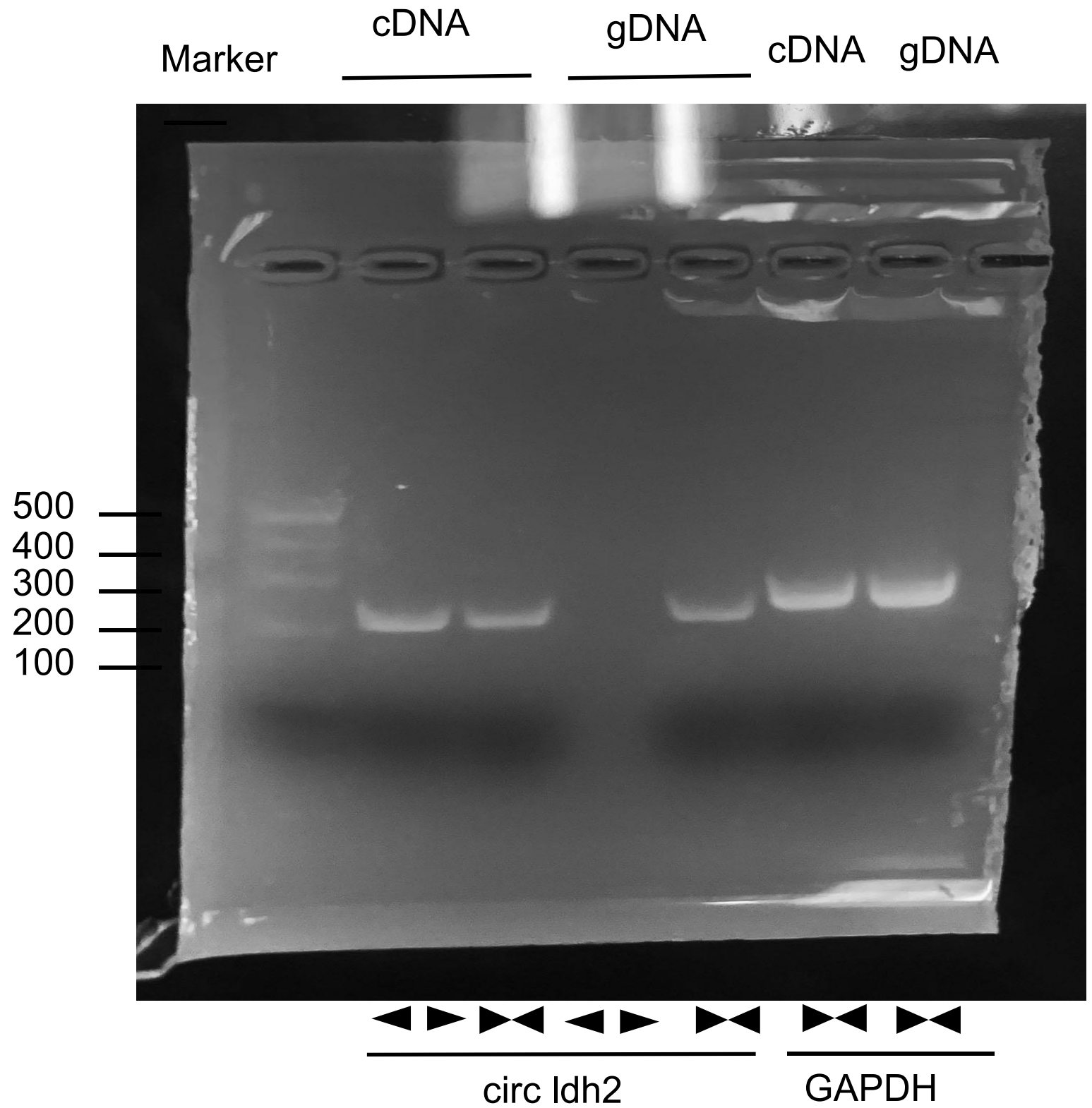

Marker
cDNA
gDNA
cDNA
gDNA
500
400
300
200
100
circ ldh2
GAPDH

Figure 2B for VE-cadherin

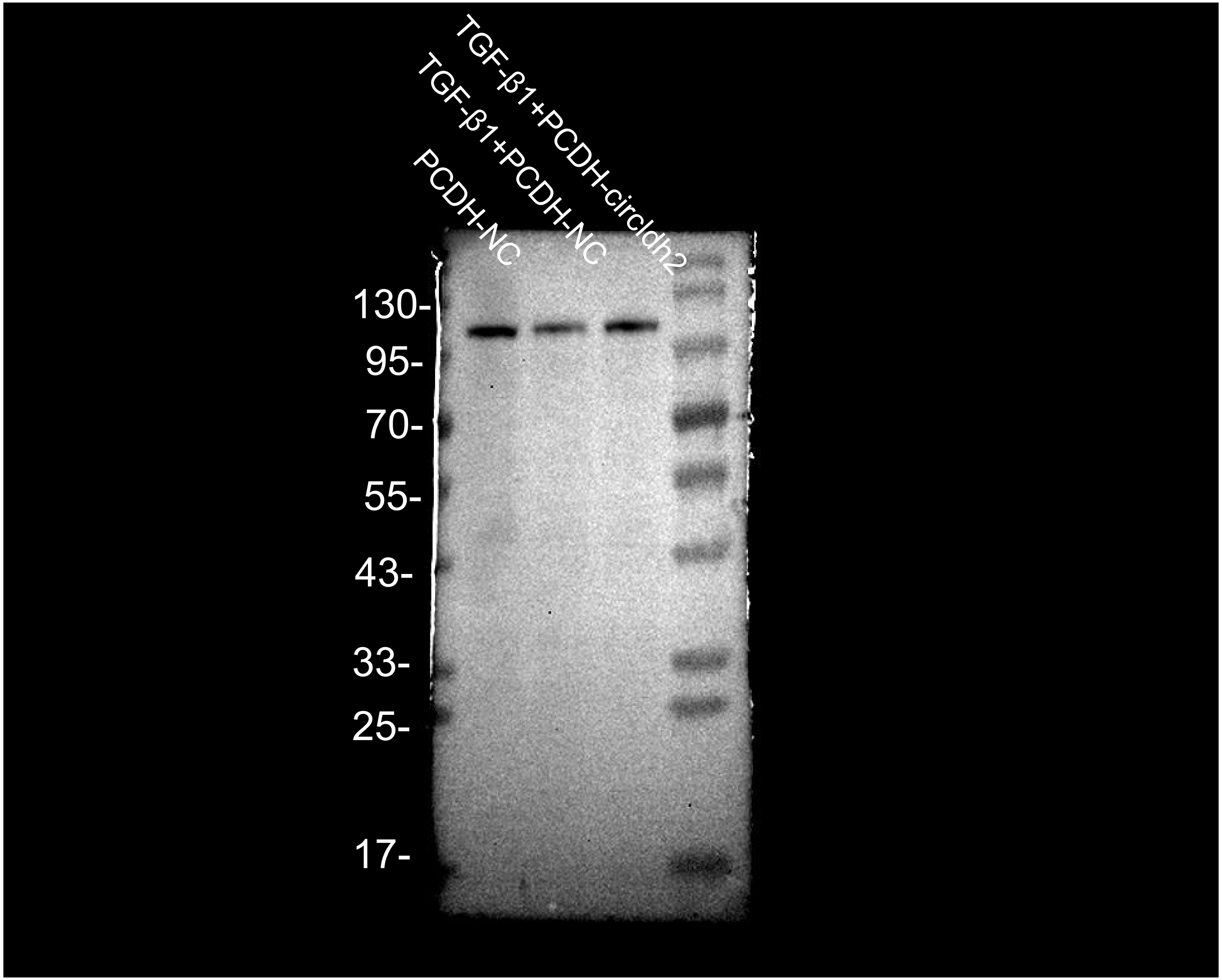

Figure 2B for Vimentin

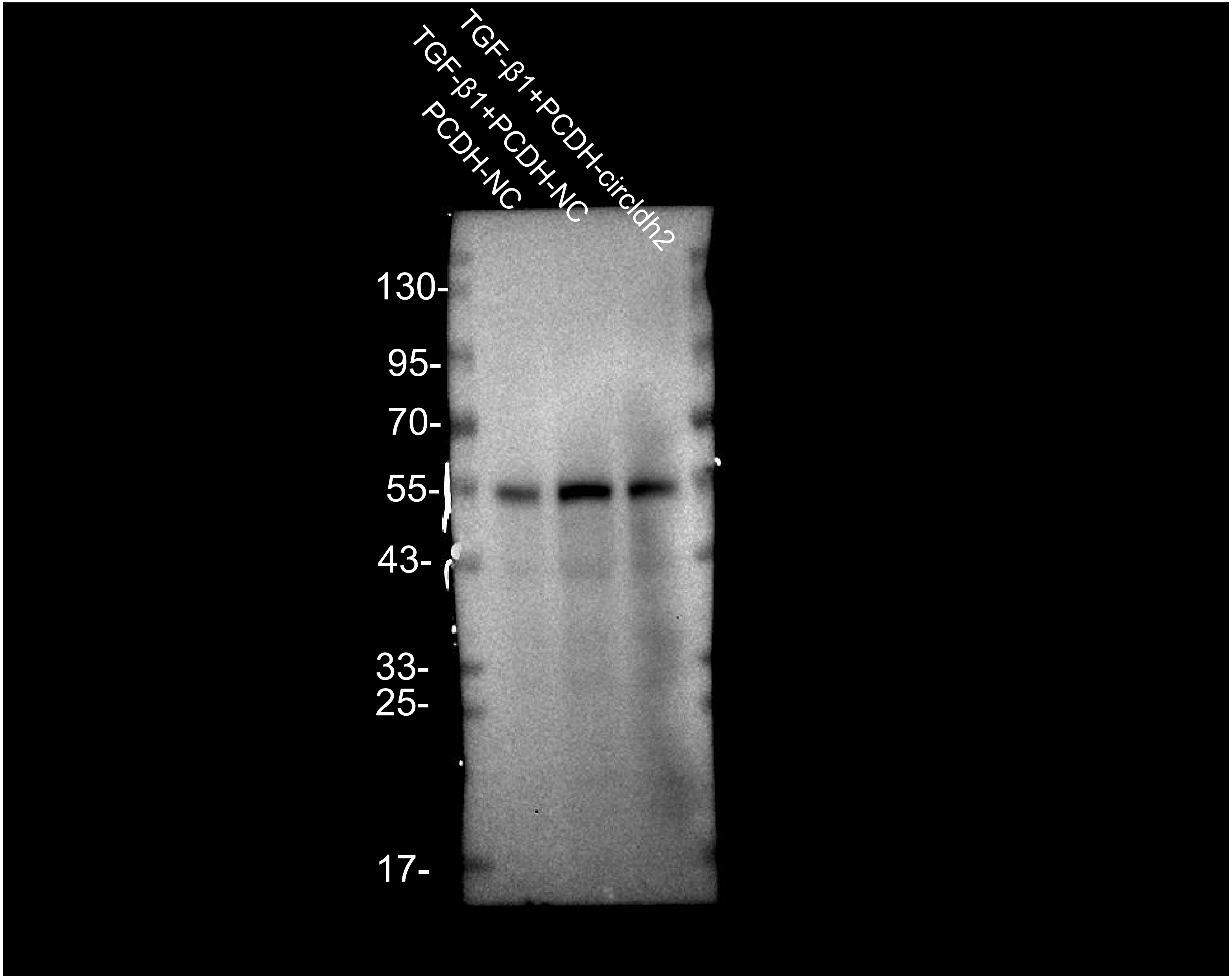

Figure 2B for N-cadherin

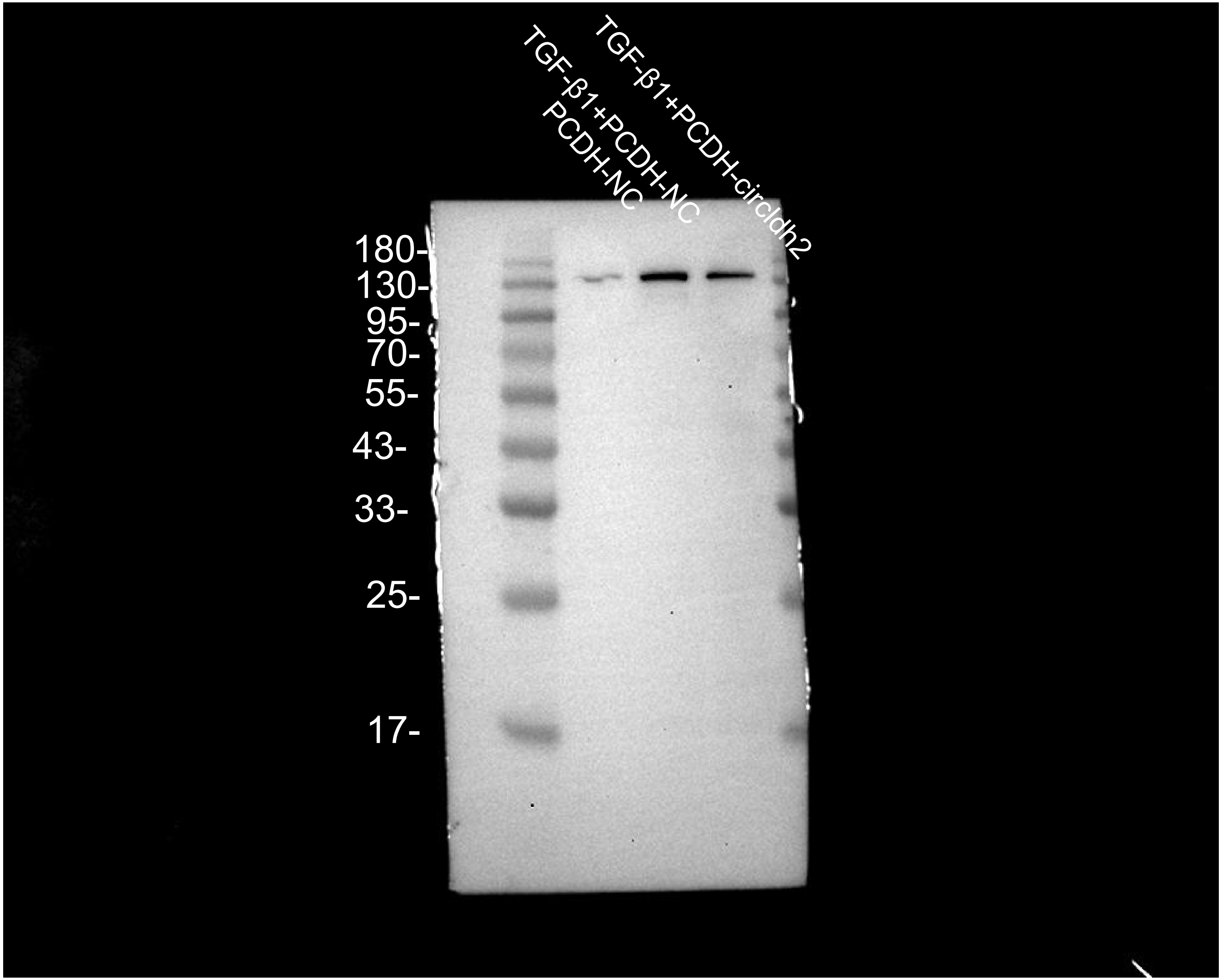

Figure 2B for GAPDH

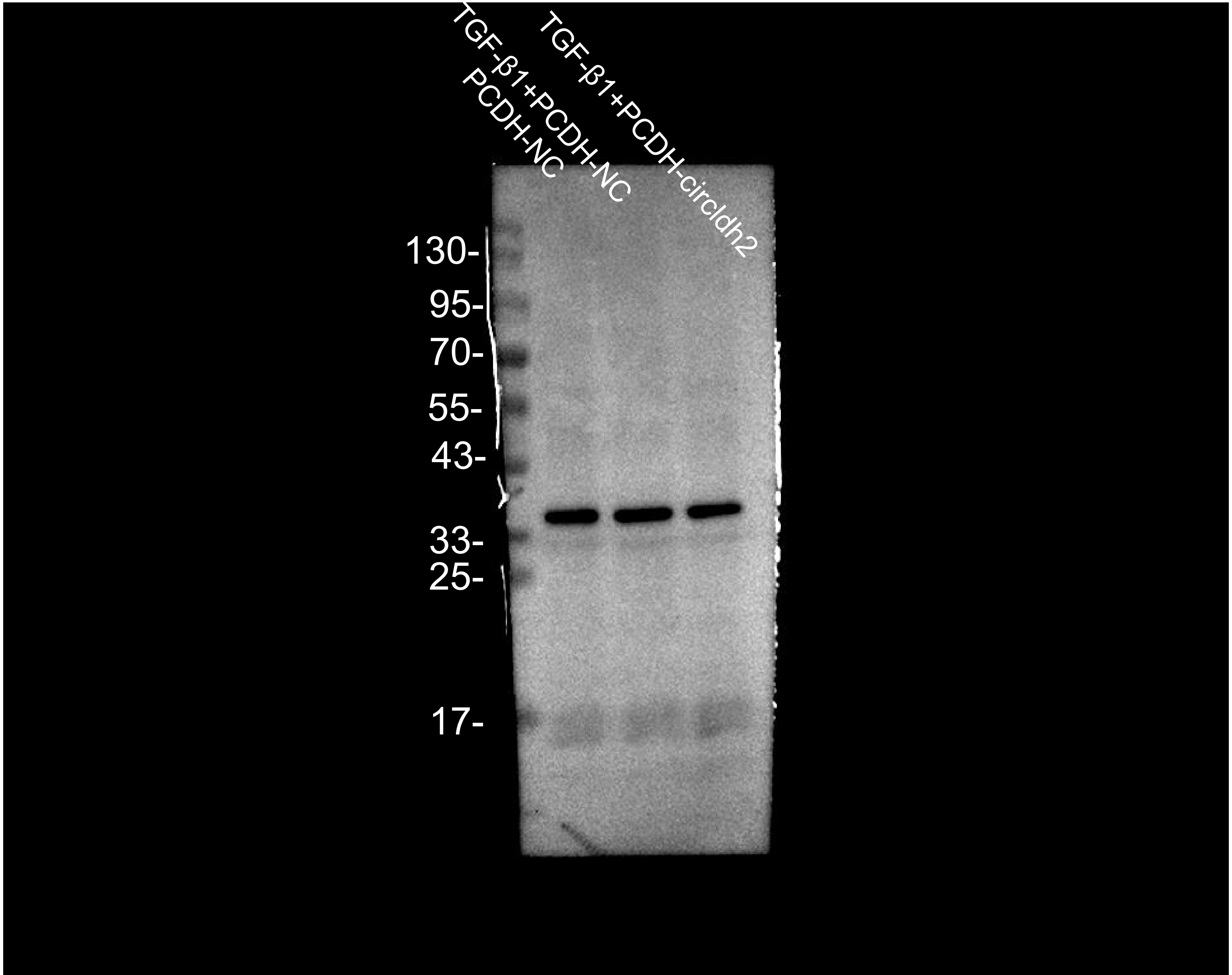

Figure 3B for VE-cadherin

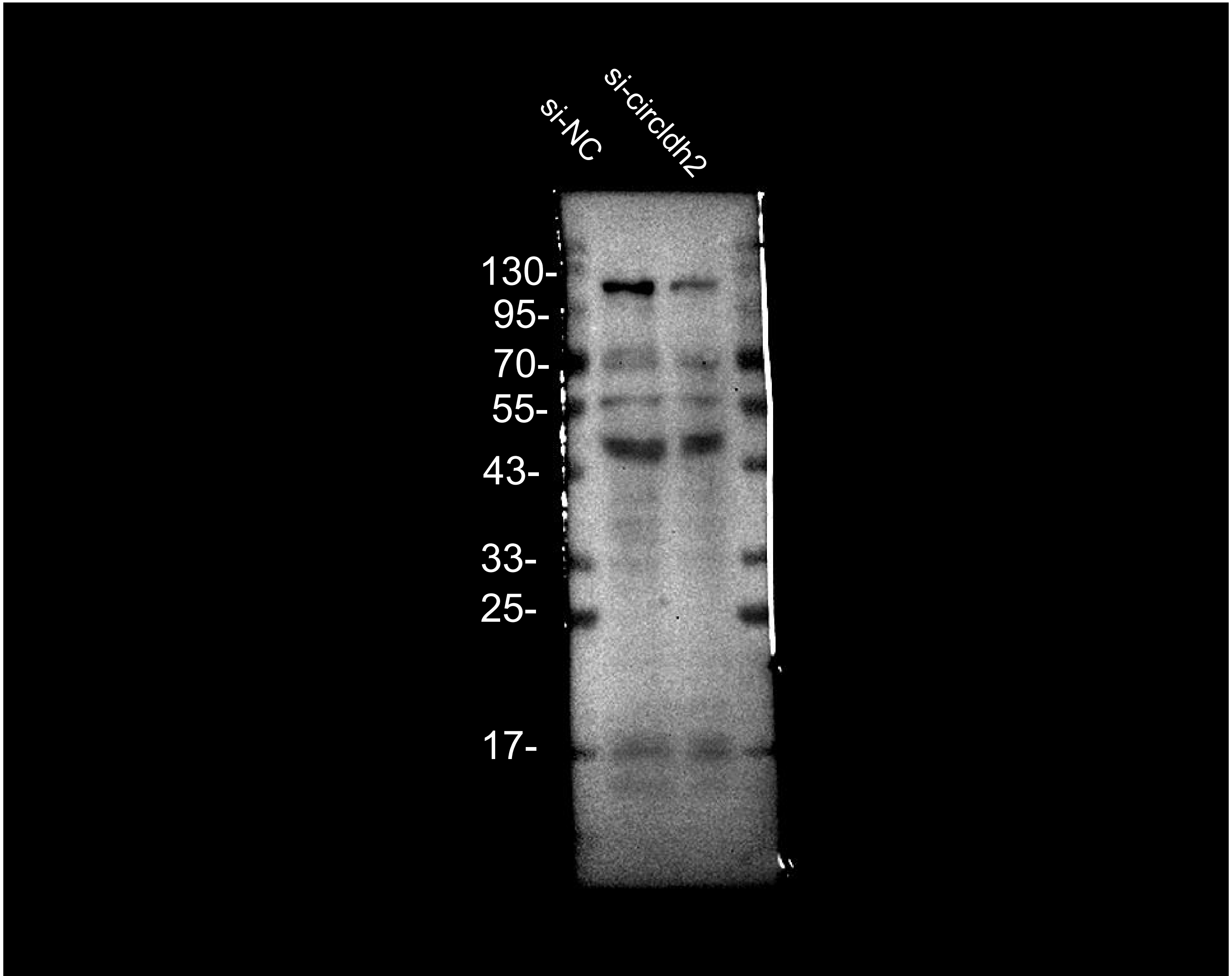

Figure 3B for N-cadherin

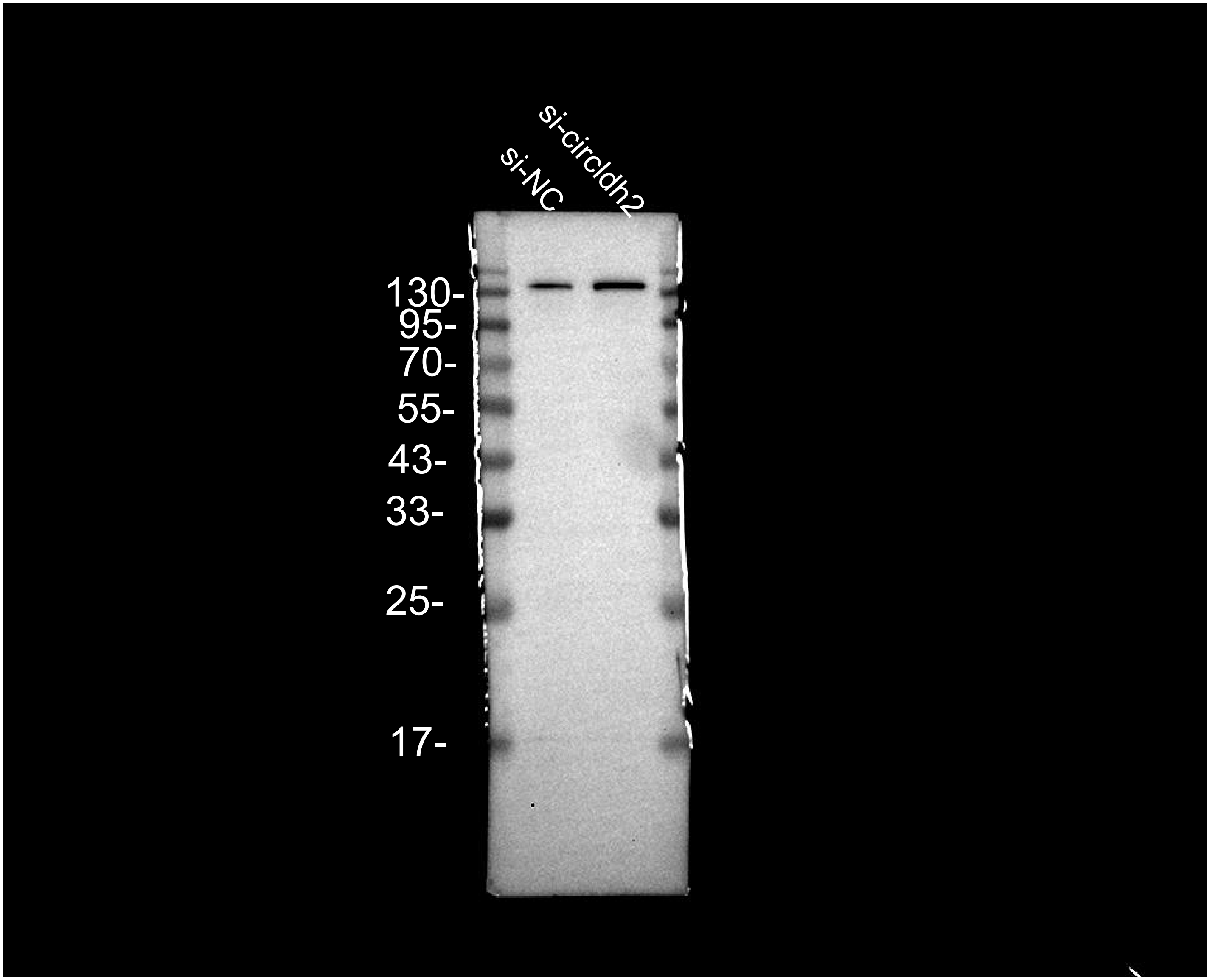

Figure 3B for Vimentin

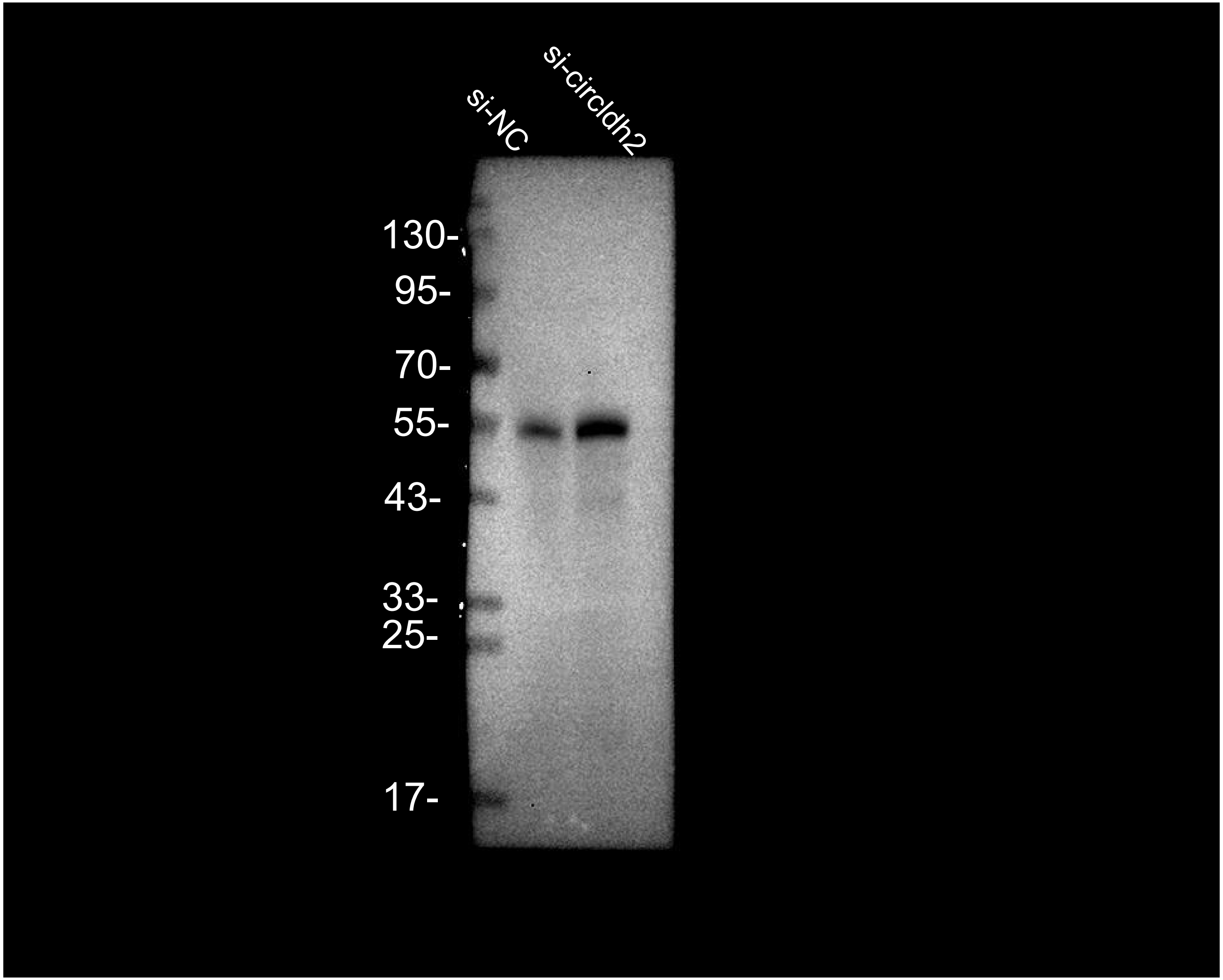

Figure 3B for GAPDH

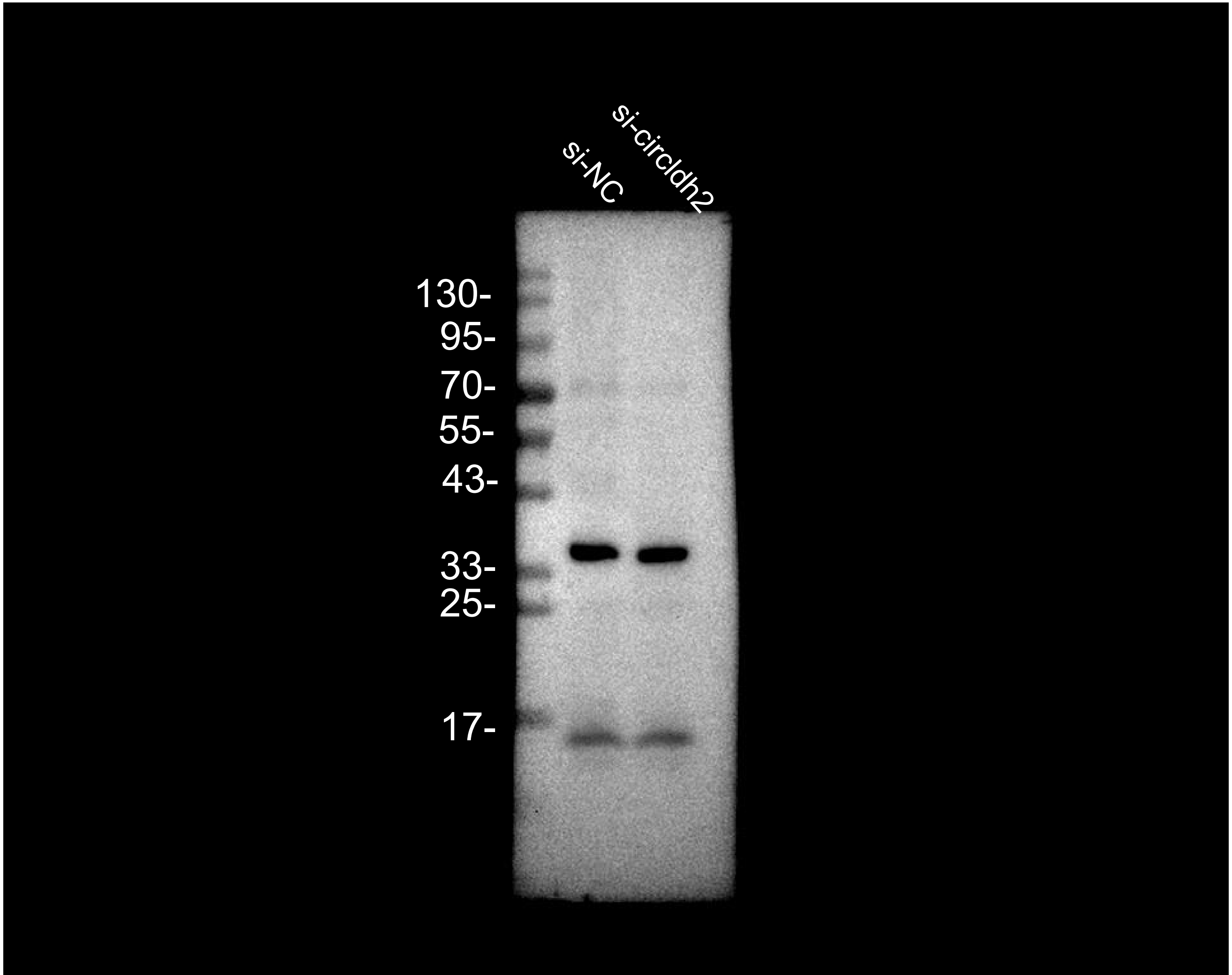

Fig 4B

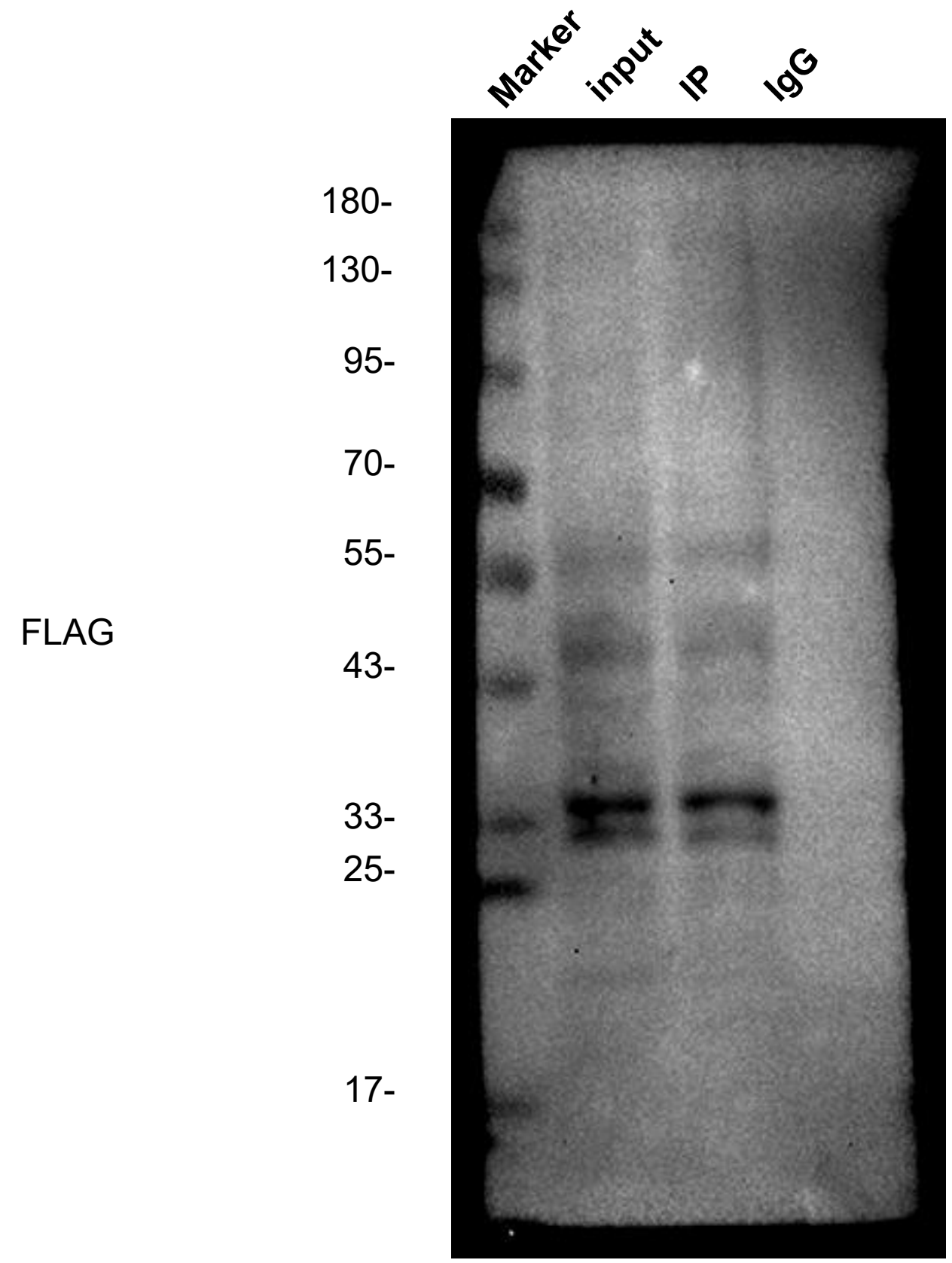

Figure 5B for flag

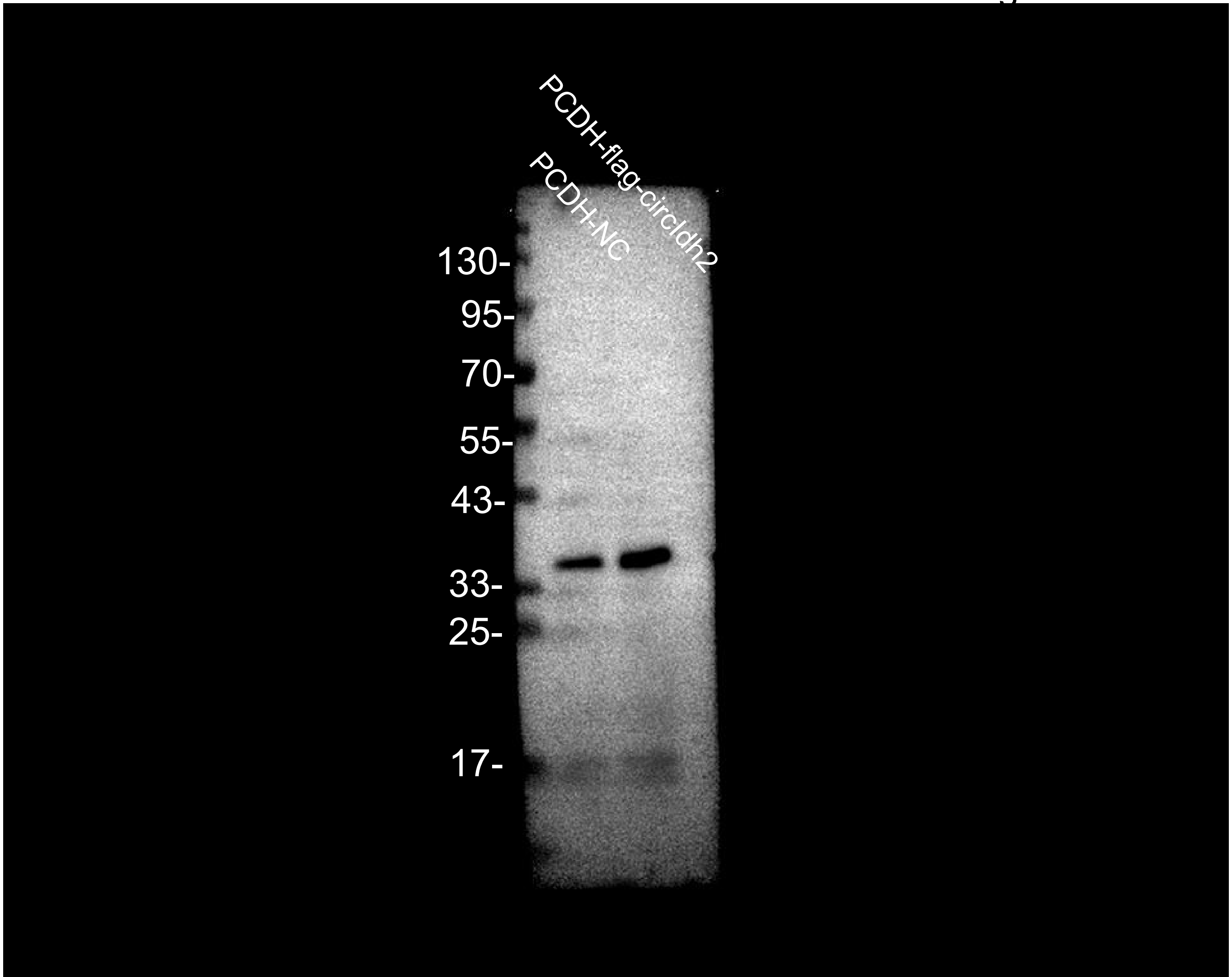

Figure 5B for GAPDH

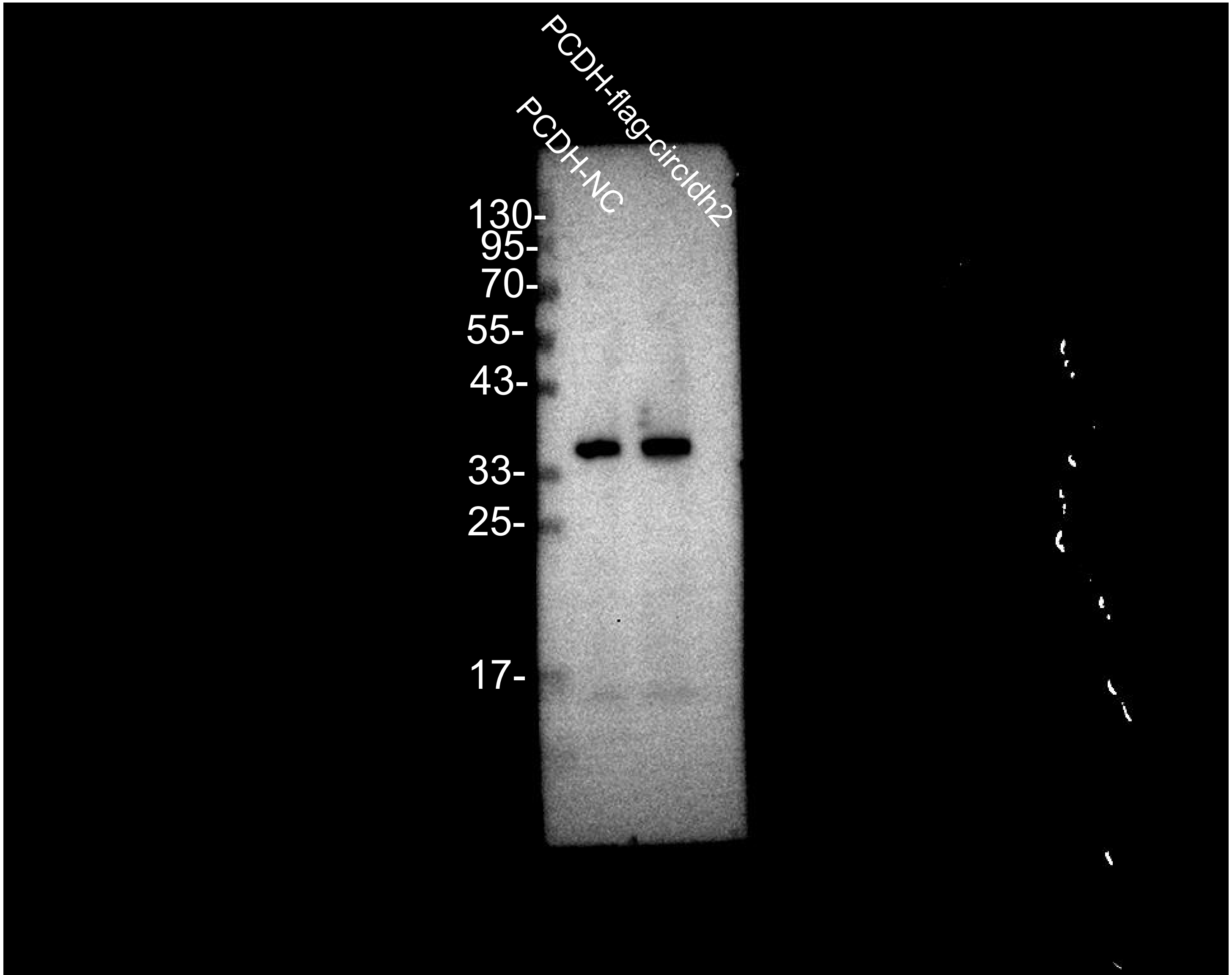

Figure 5C for VE-cadherin

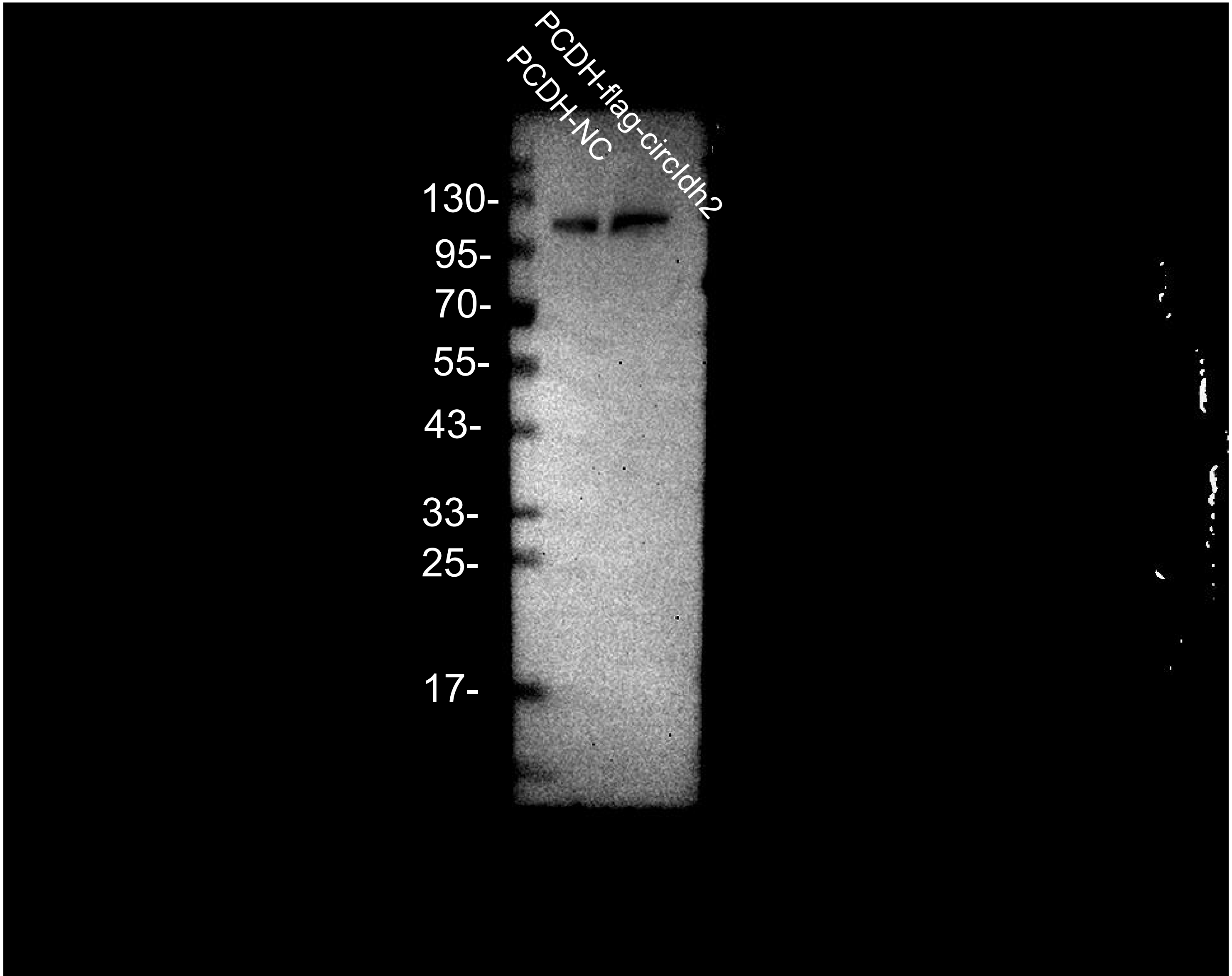

Figure 5C for Vimentin

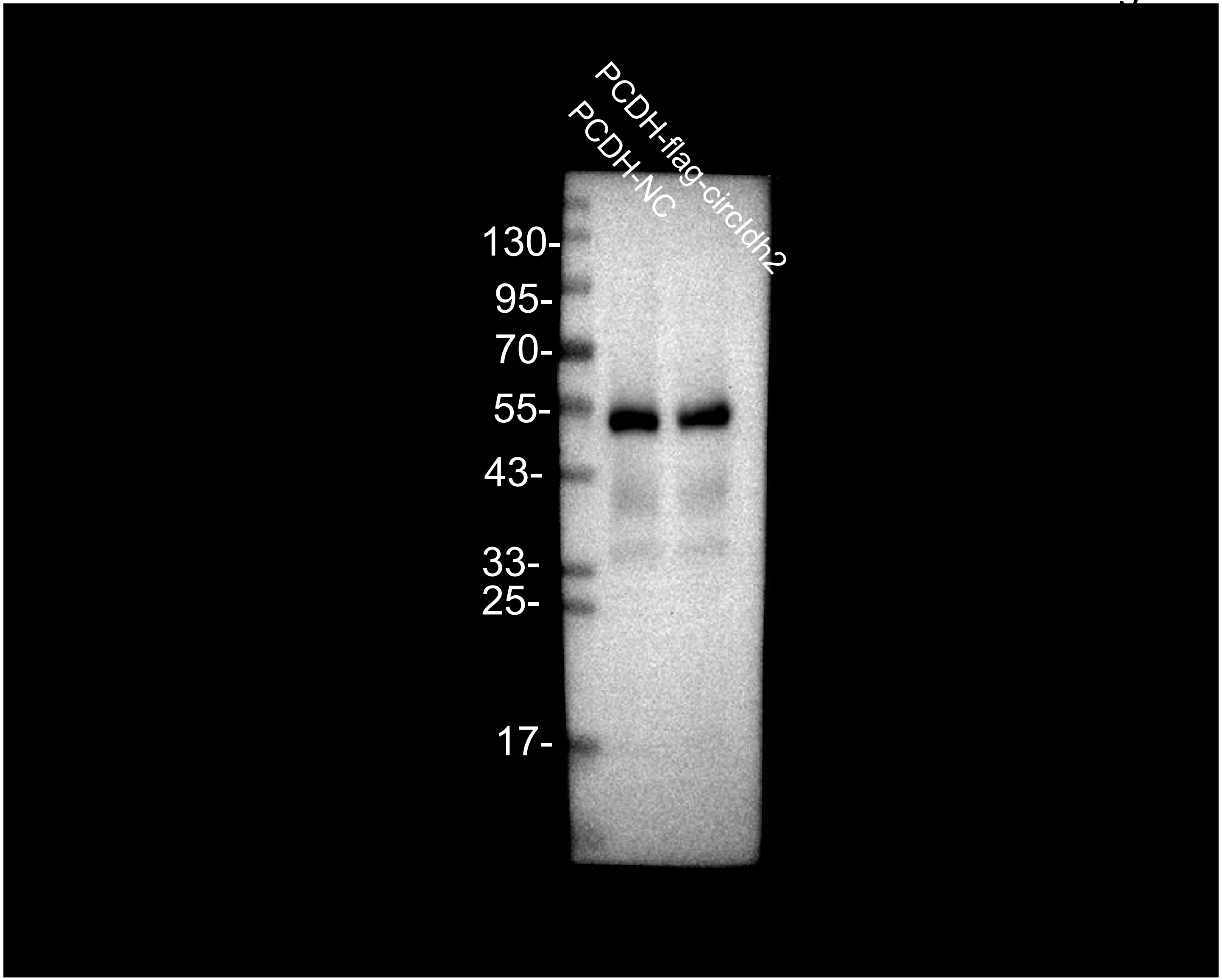

Figure 5C for N-cadherin

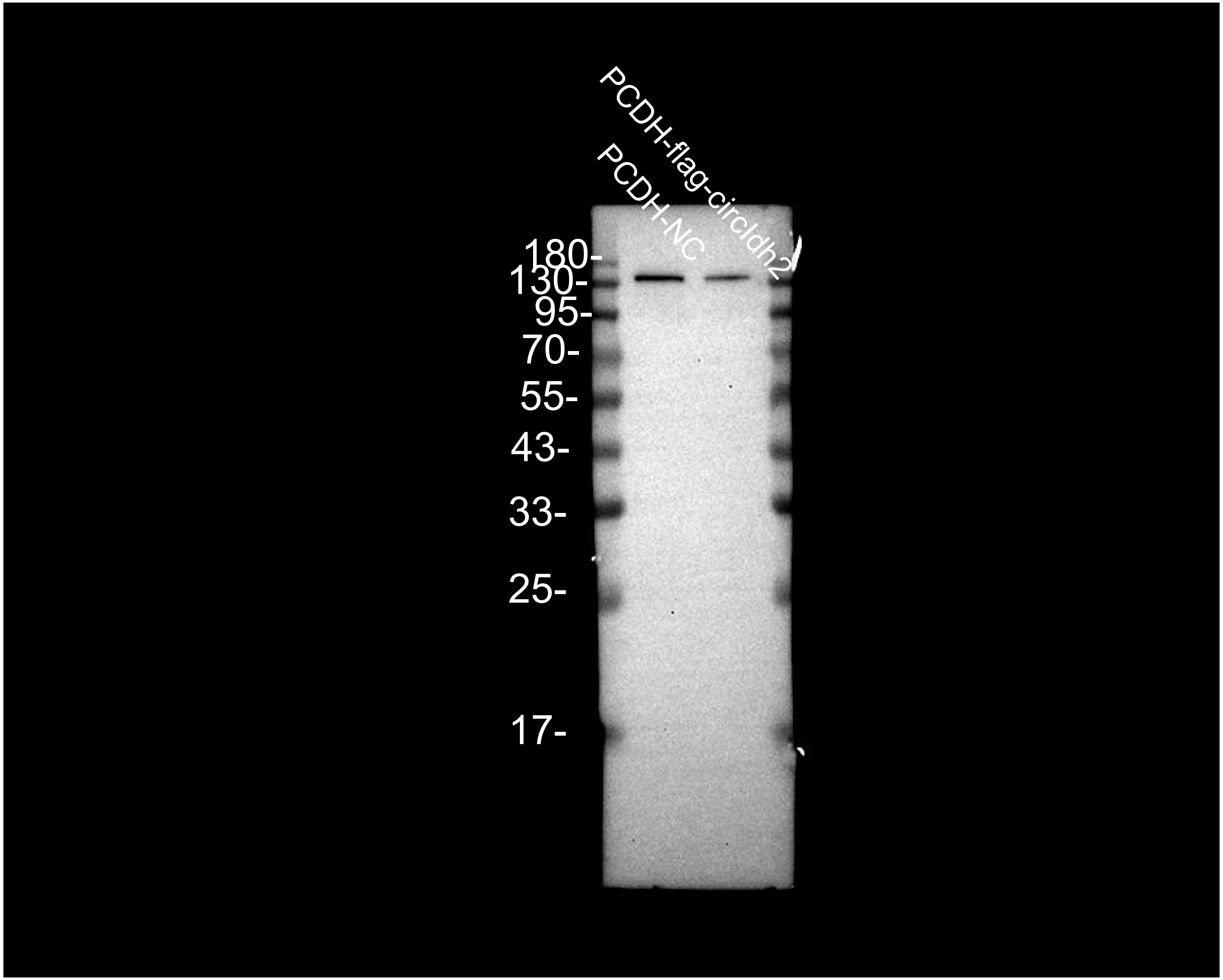

Figure 5C for GAPDH

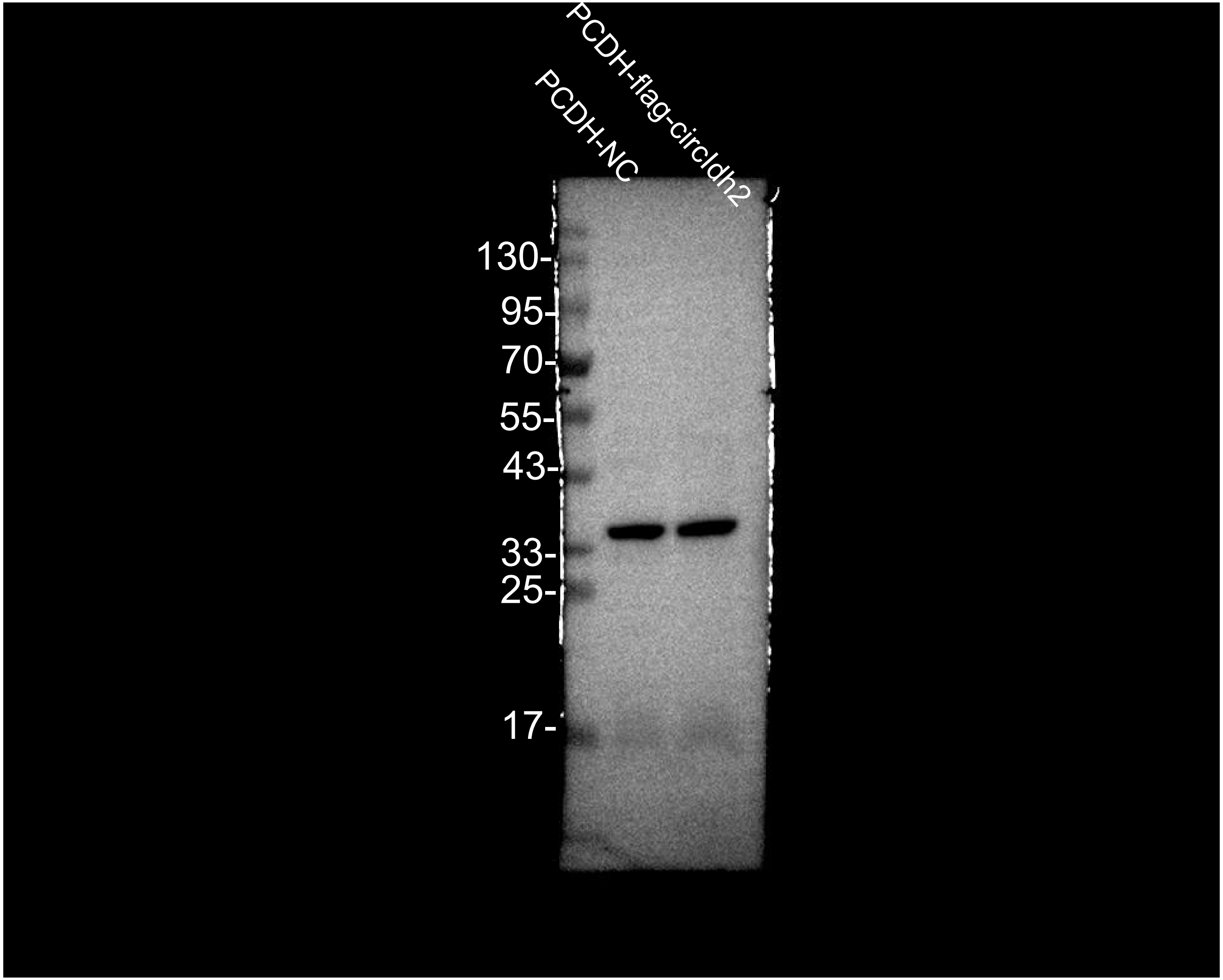

Figure 6A for VE-cadherin

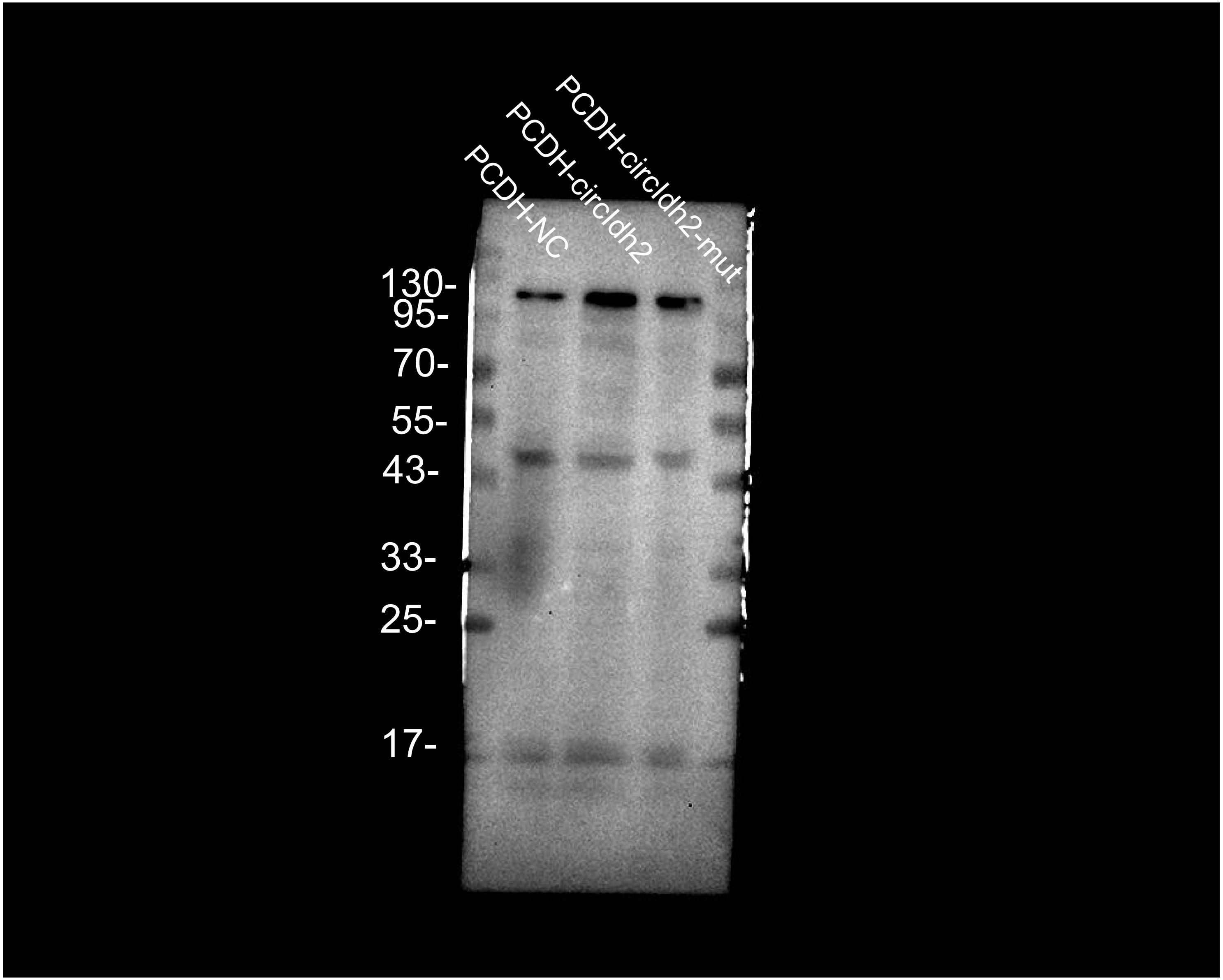

Figure 6A for Vimentin

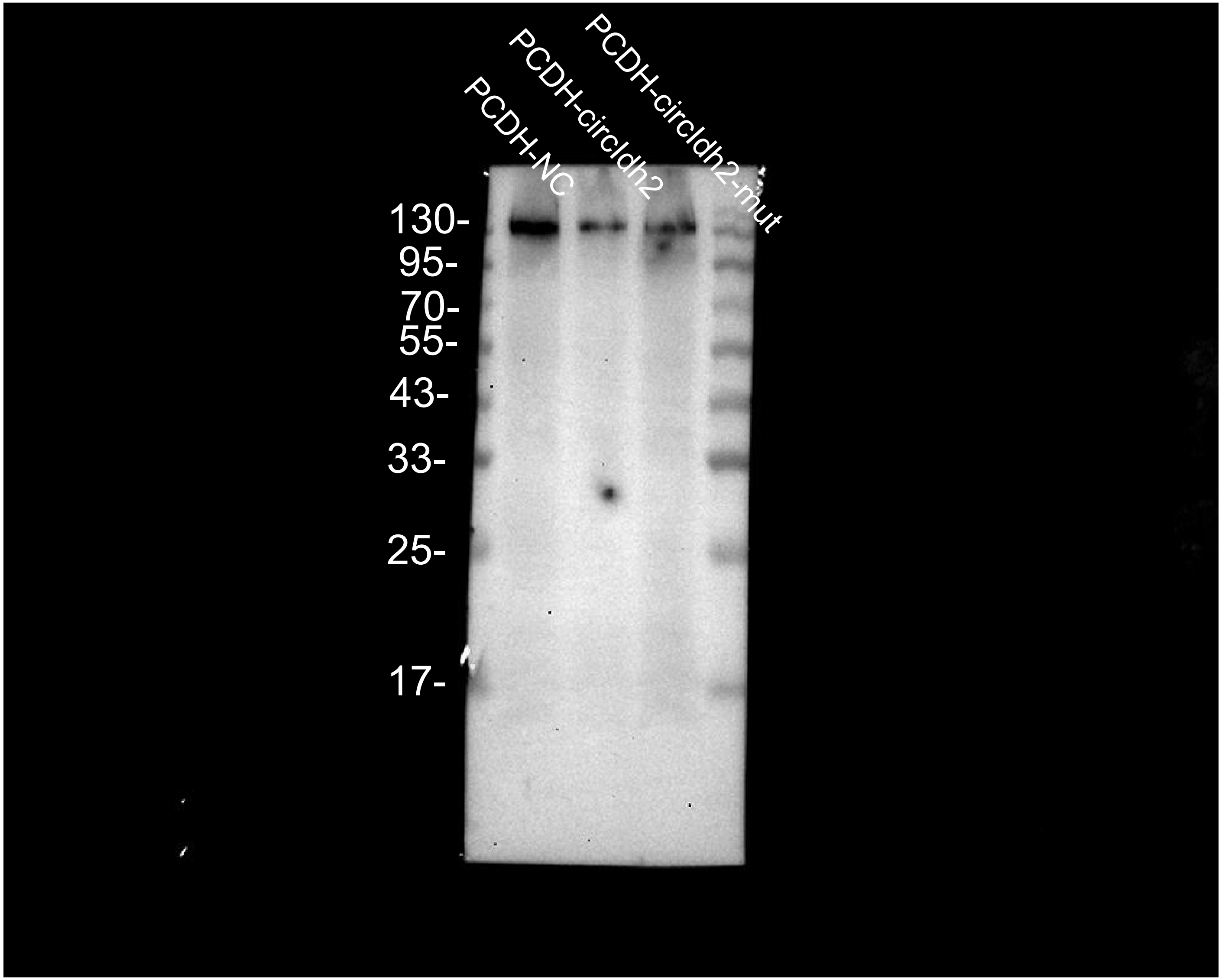

Figure 6A for N-cadherin

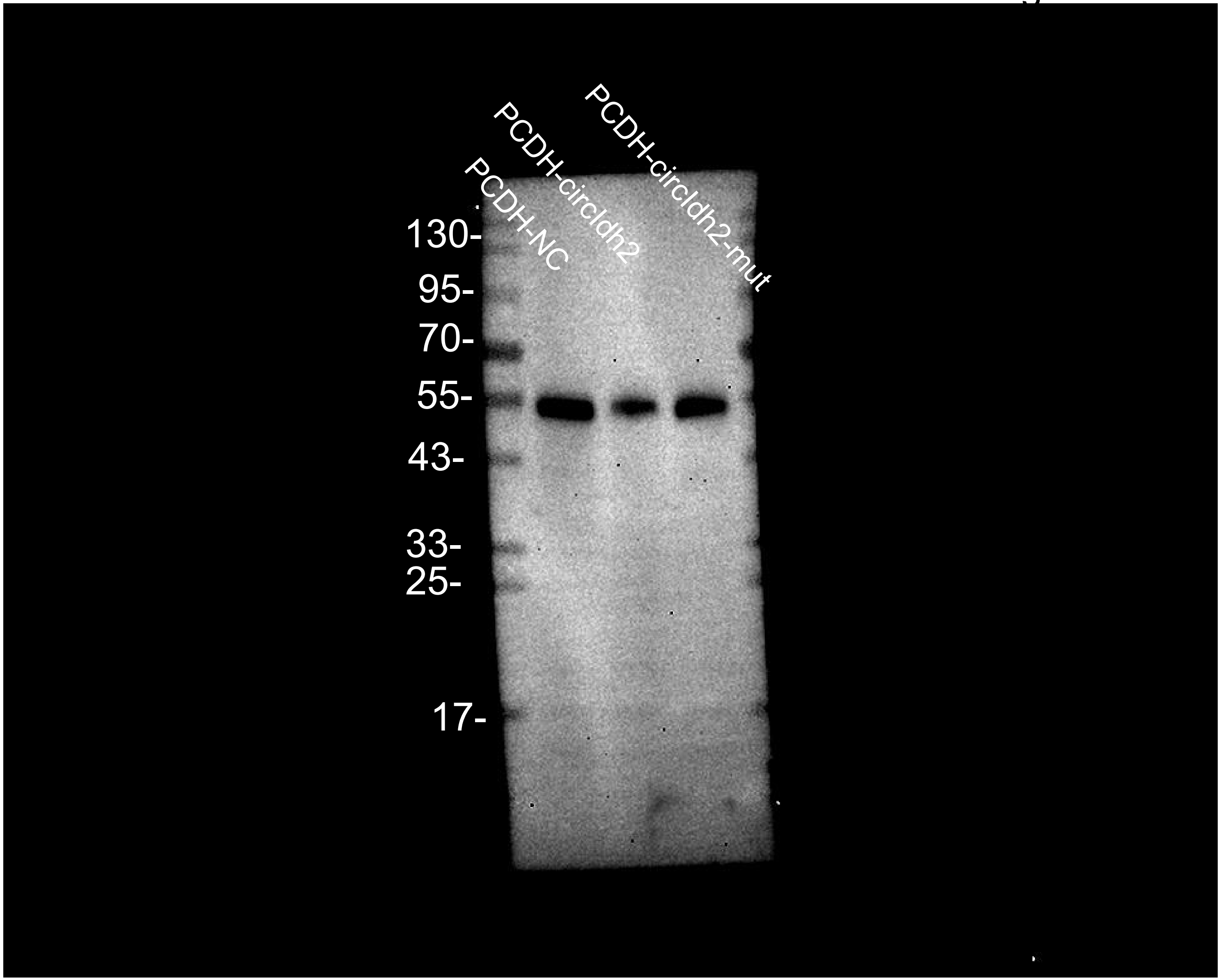

Figure 6A for GAPDH

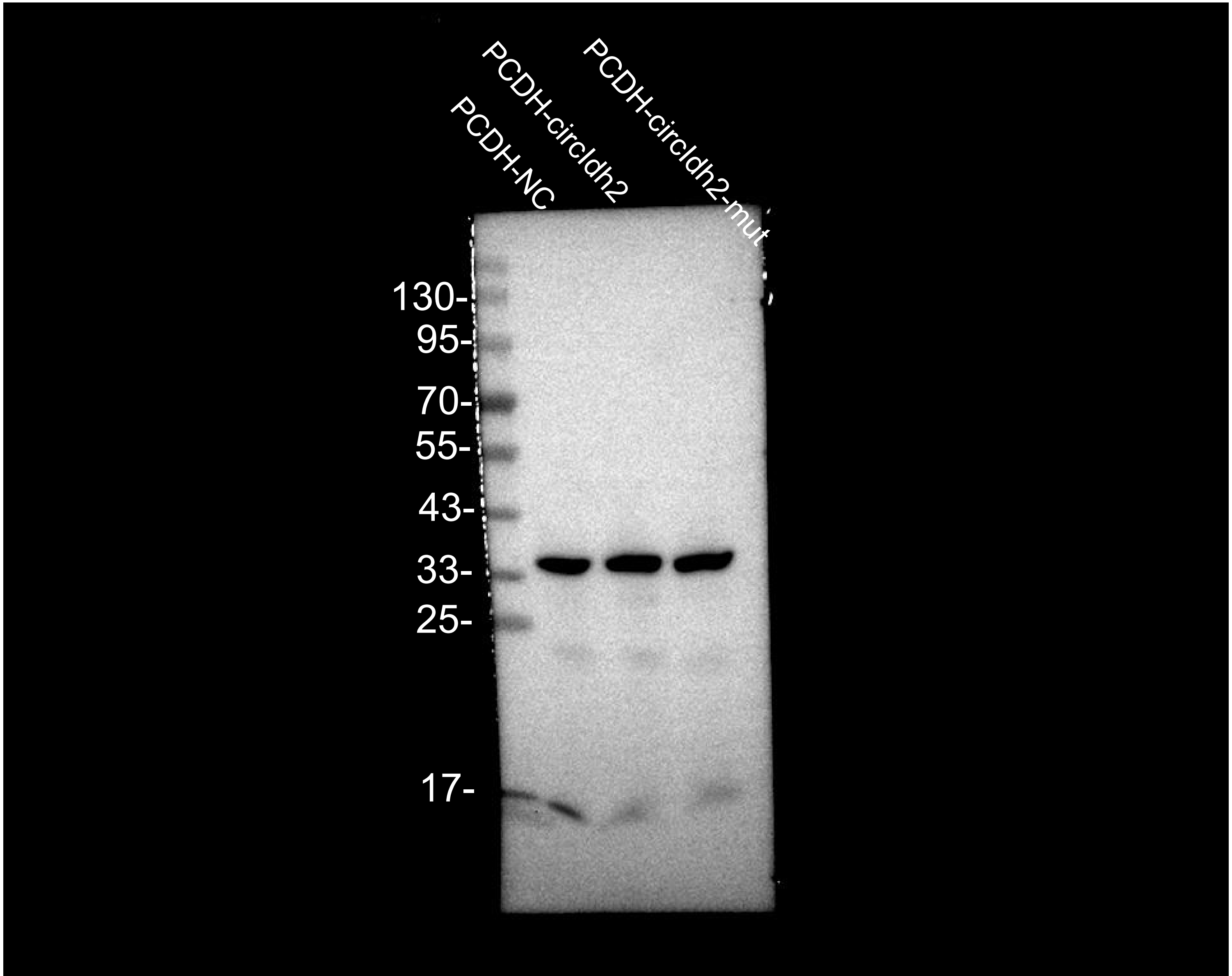

Figure 7D for VE-cadherin

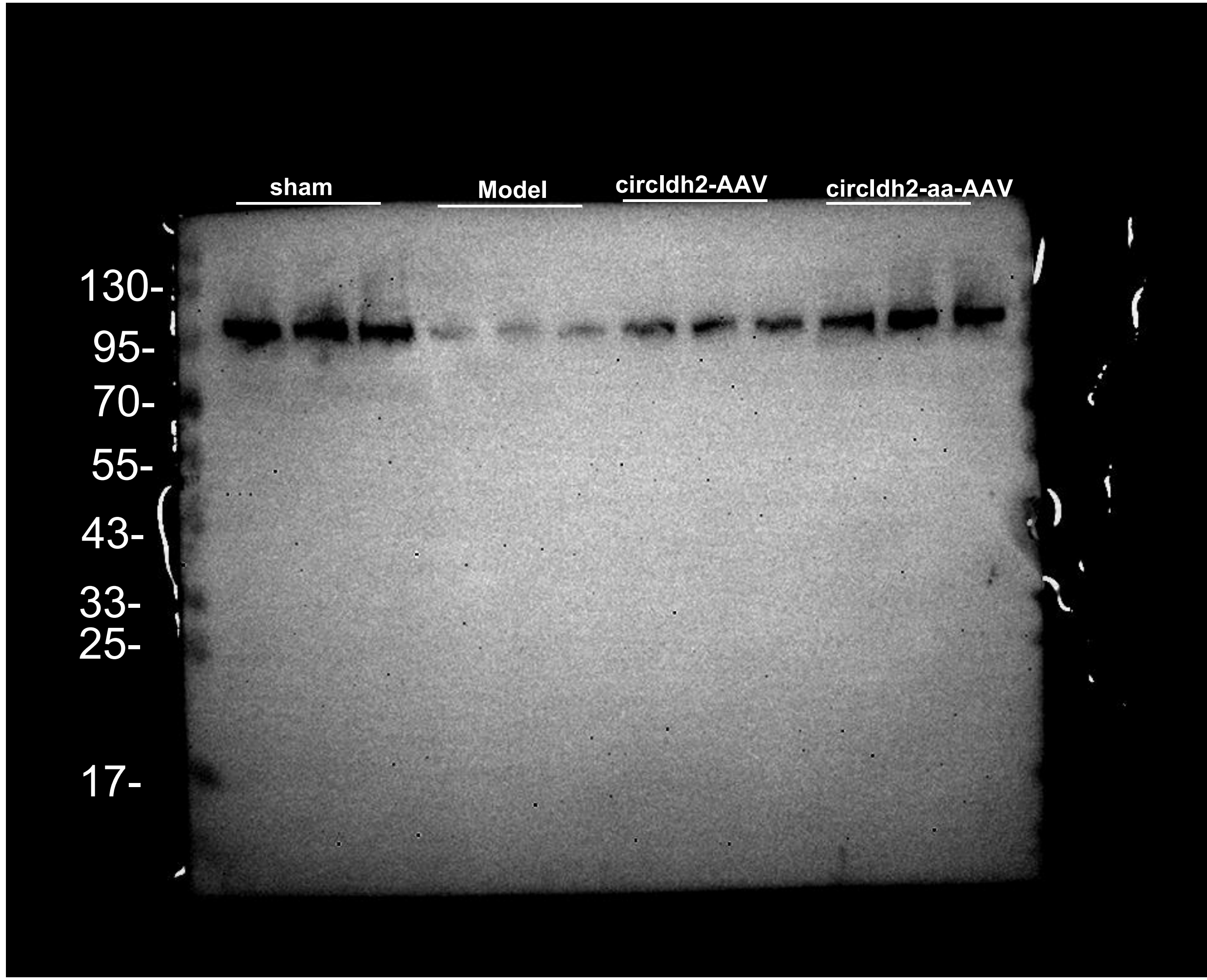

Figure 7D for Vimentin

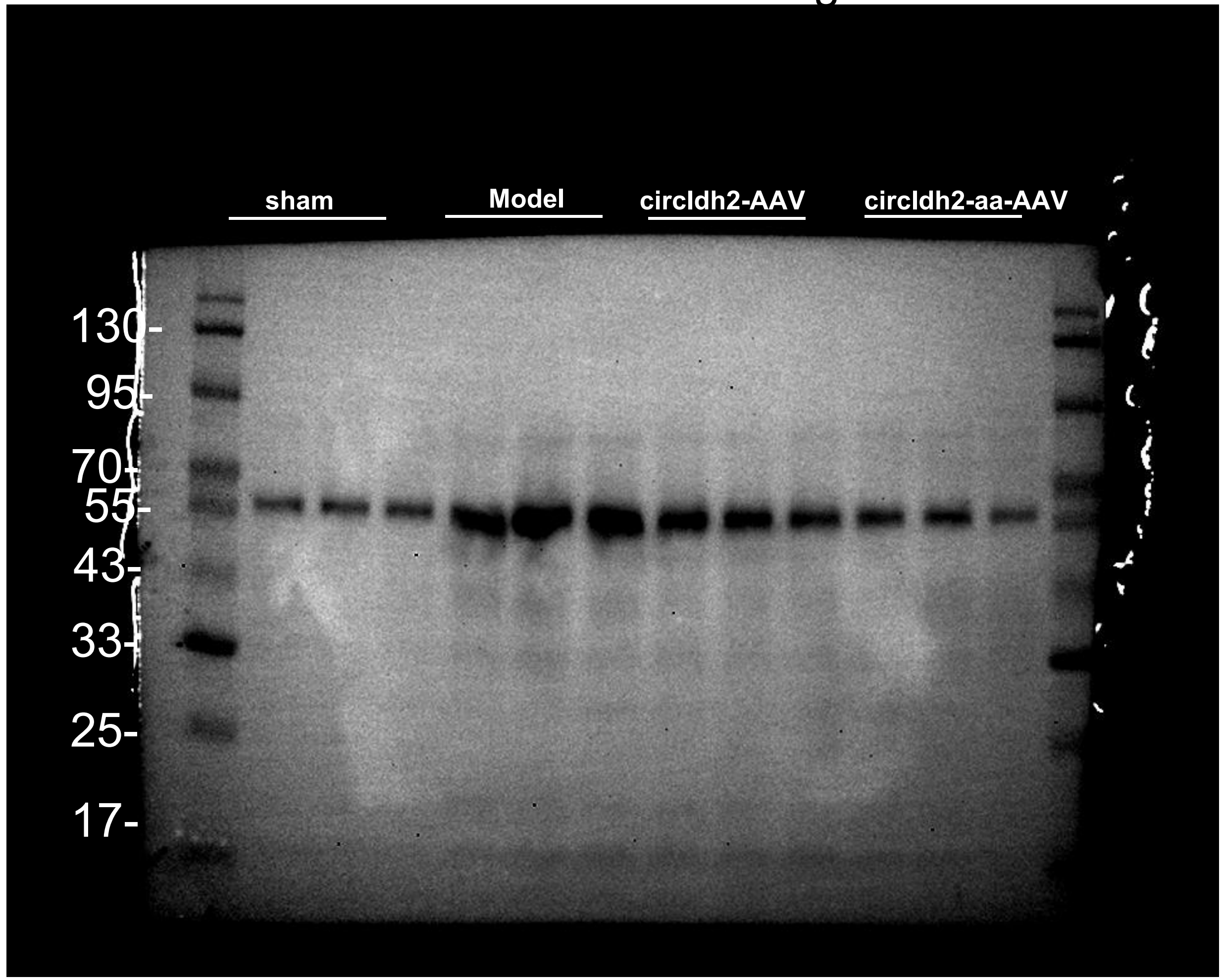

Figure 7D for N-cadherin

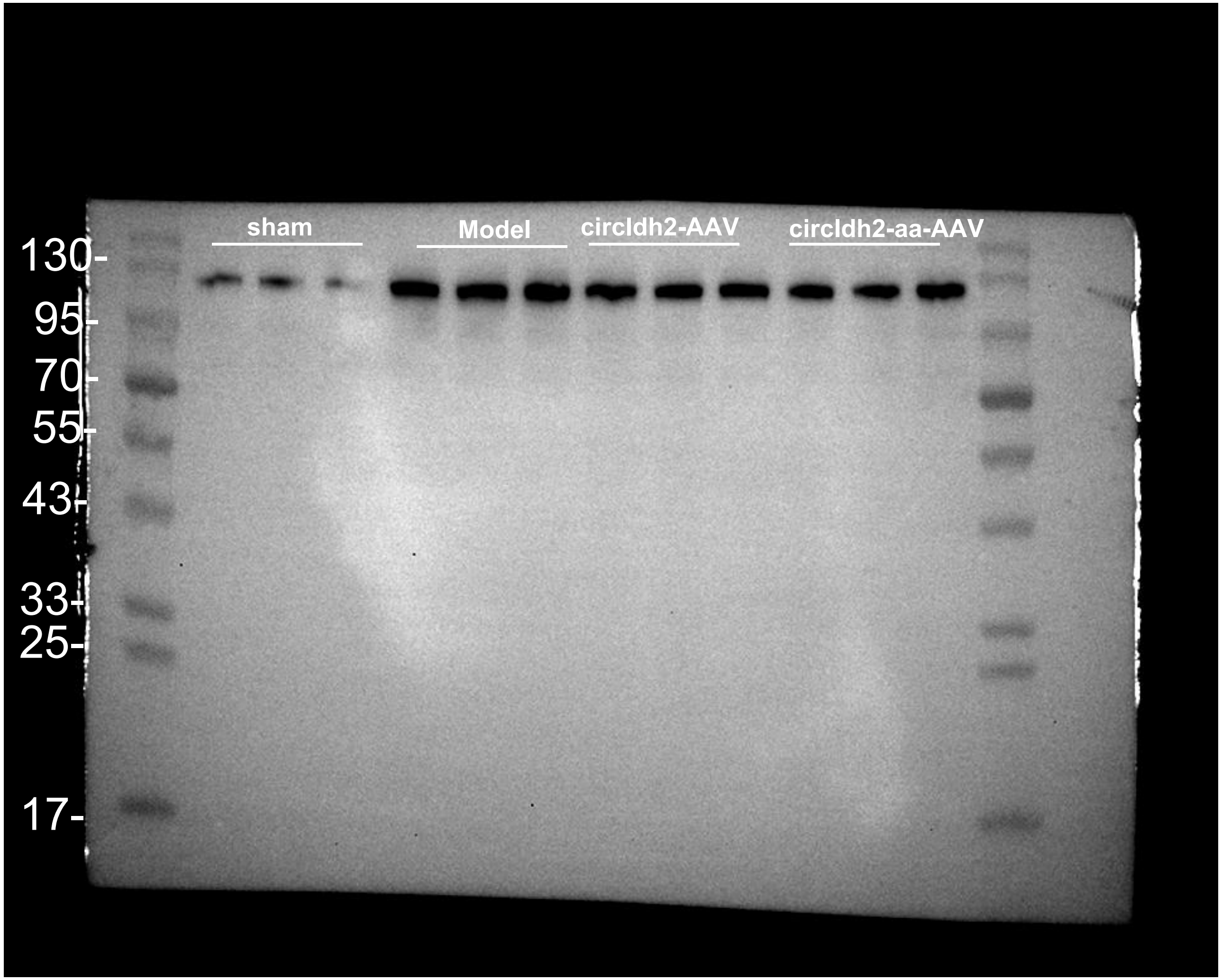

Figure 7D for GAPDH

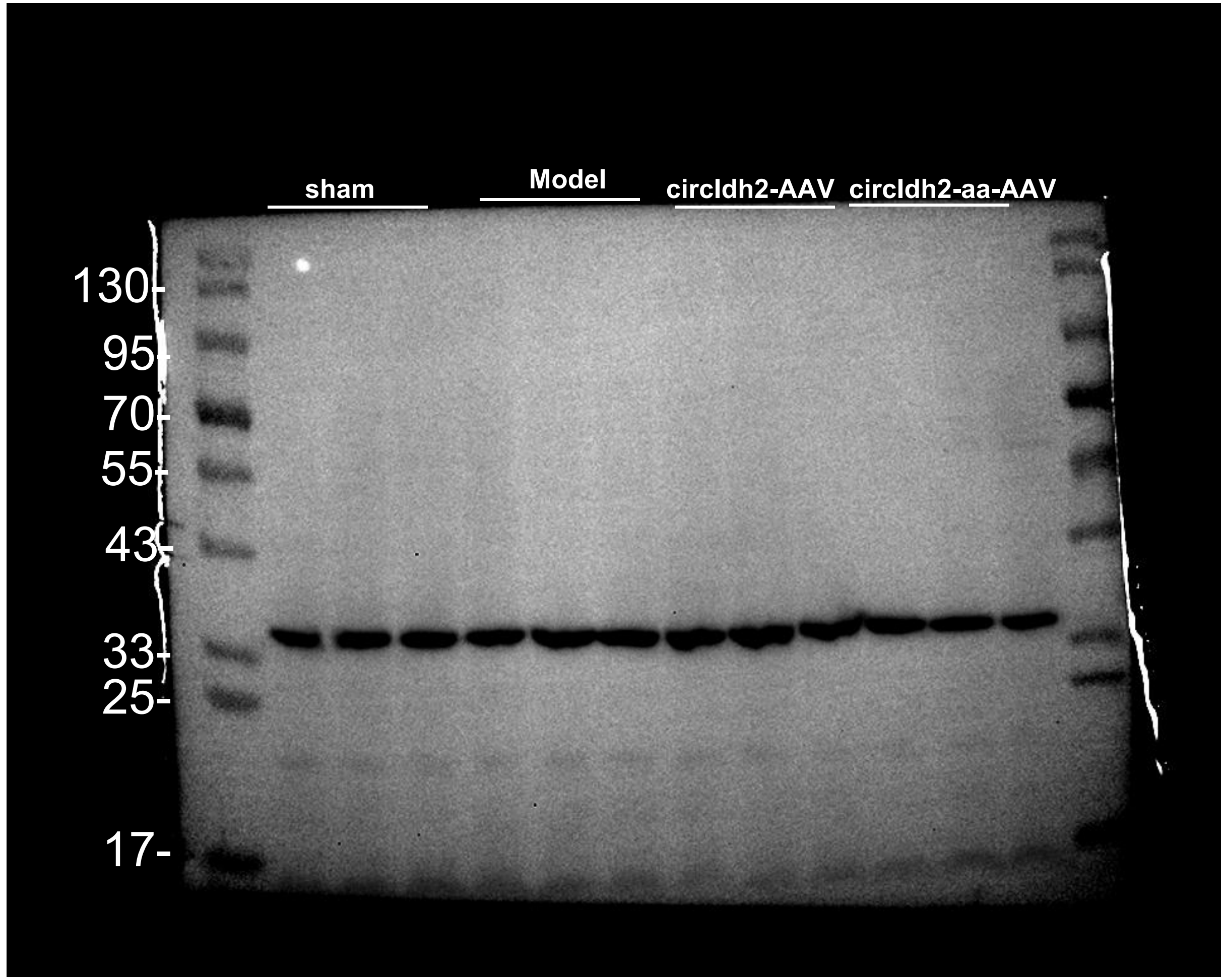

Supplement: Supplementary file 1 [file CDR-2026-2005876-s001.pdf]
