# Supplementary material for: CircIdh2 Attenuates Endothelial‐to‐Mesenchymal Transition and Myocardial Fibrosis and Exhibits Translational Potential
Source: Cardiovasc Ther. 2026 Oct 8;2026:2005876. doi: 10.1155/cdr/2005876 (PMC13647212; doi:10.1155/cdr/2005876)
Supplement: Supplementary file 2 [file CDR-2026-2005876-s002.docx]

**Supplementary Table S1**. Prespecified eligibility information and initial qRT-PCR screening results for candidate circRNAs

| **Candidate** | **Host gene** | **Human circRNA record(s)** | **Mouse circRNA record(s)** | **Rat locus (source dataset)** | **Predicted ORF** | **Baseline Ct mean ± SD** | **Baseline ΔCt mean ± SD** | **NC (2−ΔΔCt; n=3)** | **TGF-β1 (2−ΔΔCt; n=3)** | **Adjusted P value** |
| --- | --- | --- | --- | --- | --- | --- | --- | --- | --- | --- |
| circSorbs1 | Sorbs1 | hsa_circ_0094610; hsa_circ_0094609 | mmu_circ_0007771; mmu_circ_0007772 | chr1:259396273–259437470 | Yes | 22.86 ± 0.12 | 7.49 ± 0.18 | 1.00, 0.96, 0.85 | 0.45, 0.40, 0.50 | <0.0001 |
| circIdh2 | Idh2 | hsa_circ_0057970 | mmu_circ_0001587 | chr1:141877382–141880461 | Yes | 20.26 ± 0.16 | 4.89 ± 0.33 | 1.00, 0.94, 1.16 | 0.16, 0.18, 0.14 | <0.0001 |
| circSntb2 | Sntb2 | hsa_circ_0005733; hsa_circ_0105819; hsa_circ_0040039 | mmu_circ_0014531 | chr19:39176540–39195019 | Yes | 18.86 ± 0.19 | 3.50 ± 0.14 | 1.00, 1.04, 0.81 | 0.33, 0.38, 0.39 | <0.0001 |

Notes: n=3 independent biological experiments. Relative expression was calculated using the 2−ΔΔCt method with candidate-specific NC calibrators. Human and mouse IDs represent circRNA records from orthologous host genes and do not establish conservation of an identical back-splice junction. Rat loci and ORF predictions were obtained from reference 16.
